# SARS-CoV-2 and neutralizing antibody dynamics at a remote village in Kenya before widespread COVID-19 vaccine rollout

**DOI:** 10.1371/journal.pgph.0006706

**Published:** 2026-07-24

**Authors:** Esther Omuseni, Josphat Nyataya, Peter Sifuna, George Awinda, Allan Lemtudo, Clement Masakhwe, Beth Mutai, Geoffrey Maina, Simon Kariuki, John Waitumbi

**Affiliations:** 1 Kenya Medical Research Institute/Walter Reed Army Institute of Research-Africa, Basic Science Laboratory, Kisumu, Kenya; 2 Kenya Medical Research Institute, Center Biotechnology Research and Development, Nairobi, Kenya; 3 Kenya Medical Research Institute, Center for Global Health Research, Kisumu, Kenya; University of Michigan, UNITED STATES OF AMERICA

## Abstract

Given limited data on SARS‑CoV‑2 transmission and immunity in rural, low‑vaccine‑coverage settings, we evaluated infection rates and population‑level neutralizing antibody increase in a rural community in western Kenya. The study was conducted at the KEMRI/WRAIR-Africa Health Demographic Surveillance System that has a population of 160,000. Clusters of households that had representative demographics were identified and individuals invited to participate in six cross-sectional surveys that targeted 234 participants at each survey, two months apart, conducted between April 2021 and March 2022, during which time, the Alpha, Delta and Omicron waves dominated. At each survey, participants provided naso-pharyngeal samples for SARS-CoV-2 detection and serum for measuring neutralizing antibodies (NAbs) using surrogate virus neutralizing test which assessed inhibition of RBD–ACE2 binding. 69% (917/1329) of the participants were females. By RT-qPCR, SARS-CoV-2 increased from 3% (7/224) at survey one (April 2021) to 24% (55/229) by survey two (June 2021), and thereafter decreased to zero, only to rise to 10% (23/234) during the January 2022 Omicron wave. NAbs increased from 18% (40/224) at survey one to 86% (202/234) by end of the study. The quantity of NAbs rose from geometric mean of 0.42 U/mL at survey one to 9.4 U/mL by survey six. Females had higher NAbs (3.73U/mL, 95% CI), compared to 1.98 U/mL, 95% CI (p = 0.0146) for males. NAbs quantities increased with age: 2.1 U/mL for <9 years, 2.6 U/mL for 10 – 19 years, 3.4 U/mL for 20 – 39 years, 3.8 U/mL for 40 – 59 years and 4.9 U/mL for >60 years. The increase in population-level NAbs is consistent with widespread exposure to the virus during the study period. As caveat, causality of NAbs for the decline in SARS‑CoV‑2 infections cannot be inferred with confidence, because other factors such as the use of non‑pharmaceutical interventions and other individual-level variables were not measured.

## Introduction

The coronavirus disease 2019 (COVID-19) pandemic was caused by an emergent beta-coronavirus (SARS-COV-2) [1] that first appeared in Wuhan, China in December 2019 [2]. The virus rapidly spread globally and on 11 March 2020, the World Health Organization declared the disease a global pandemic [3]. By the time COVID-19 was declared no longer “a public health emergency of international concern” in March 2023, it had infected about 765 million people globally and killed nearly 7 million. Of these deaths, only 174,211 were reported from Africa [4]. In Kenya, the first case was traced to a Kenyan citizen who arrived in Nairobi from the United States on 5^th^ March 2020 [5]. By March 2023, there had been 342,967 confirmed cases of COVID-19 and 5,689 deaths [6]. These numbers are a gross underestimate since the biggest majority of infected people exhibit mild signs of the disease; therefore, the fraction of asymptomatic infections that did not seek medical attention are unaccounted.

Like other coronaviruses, SARS-CoV-2 contains four structural proteins that include spike (S), envelope (E), membrane (M) and nucleocapsid (N). The virus uses the spike protein to bind to the host cell-surface receptor through the Receptor Binding Domain (RBD) protein in the S1 subunit, followed by fusion of the S2 subunit to the cell membrane during host cell entry [7]. In addition to playing a role in determining the viral host range, and infectivity, the S protein is a critical target for inducing SARS-CoV-2 specific NAbs that correlate to immunity [1]. NAbs are crucial for viral clearance and protection against SARS-CoV-2 by interfering with virion binding to RBD. Measurement of NAb to RBD provides information on the magnitude of immune response resulting from disease and vaccination [8].

Neutralizing activity can be determined in multiple ways, including plaque reduction neutralization test (PRNT) [9] using wild-type virus, pseudovirus (pVNA) [10] and ELISA-based binding assays using surrogate virus neutralization tests (sVNT) [11]. Although the PRNT is the gold standard for measurement of neutralizing activity, the assay is labor-Intensive, time-consuming and can have high variability depending on cell line, virus strain, operator competence and requires BSL3. pVNA has similar drawbacks as the PRNT. Although the ELISA-based antibody binding assays are easier, they measure RBD–ACE2 binding inhibition as a surrogate to neutralization. Several ELISA-based assays and surrogate virus neutralization tests (sVNTs) have been developed to detect and quantify antibodies against SARS‑CoV‑2. These assays commonly use recombinant spike protein domains (most often the RBD) or the host receptor ACE2 as capture reagent, and an HRP‑conjugated detection reagent (e.g., ACE2‑HRP when RBD is coated, or RBD‑HRP when ACE2 is coated) to report binding or inhibition of RBD–ACE2 interaction [9]. NAbs can be raised only against S protein. This is because SARS-CoV-2 invades its host via interaction of its S protein with ACE-2 protein on the surface of host cells [12]. sVNT detects neutralizing antibodies without the need for any live virus or cells and can be completed in 1–2 hours in a BSL2 laboratory [13].

Population‑based data on SARS‑CoV‑2 transmission and immunity from rural, low‑vaccine‑coverage settings in Africa were limited during 2020–2021 and the few that existed reported multiple shortcomings. For example, a Kenyan HDSS and household cohort studies documented markedly lower seroprevalence in rural sites (e.g., Kilifi, Asembo) than in many urban samples and rapid increases in seropositivity over short intervals, while national blood‑donor surveys and continent‑wide reviews highlighted heterogeneous seroprevalence and the underrepresentation of rural populations [14–17]. Moreover, vaccine coverage across much of the African Region remained low through 2021, indicating that observed antibody increases largely reflected prior infection rather than vaccination [18,19].

Given the limited data on SARS‑CoV‑2 transmission and immunity in rural, low‑vaccine‑coverage settings, we conducted a population-level study within the WRAIR-Africa Health Demographic Surveillance System (HDSS) site in Kombewa, Western Kenya. A repeated cross-sectional design was used to characterize temporal patterns of SARS-CoV-2 infection using RT-qPCR and sVNTs responses prior to the widespread COVID-19 vaccine rollout.

## Materials and methods

### Study site and population

The study was conducted at the WRAIR-Africa’s HDSS site in Kombewa, western Kenya that has a population of about 160,000 rural inhabitants who live in about 30,000 households [20]. The primary socio-economic activities include subsistence small scale farming, livestock keeping and fishing. Kombewa was selected due to the presence of an already established HDSS population that is monitored routinely at individual household level and data on vaccinations, illness, migration, births and deaths collected.

### Ethical approval

The study was open to all age groups and gender living within the HDSS households. The study ethical approval was provided by the Kenya Medical Research Institute (KEMRI) Scientific and Ethical Research Unit (SERU) (KEMRI/SERU #4033) and the Walter Reed Army Institute of Research (WRAIR) Human Subject Protection Branch (HSPB) (WRAIR/HSPB #2813).

### Study design and sampling

This was a cross-sectional study, conducted through six surveys spaced two months apart over a period of twelve months. Repeated cross-sectional surveys were selected because they allow robust, time dependent, population level monitoring of seroprevalence trends while establishing and maintaining a large longitudinal prospective cohort was not feasible due to logistical, ethical, and resource constraints in this setting. The HDSS data manager identified clusters of households that had appropriate age band categories and individuals invited to participate in the study. Prior to participation, informed consent/assent was obtained from the participants. Consenting was done in English or the local language if desired, i.e., Kiswahili or Dholuo. Nasopharyngeal/oral-pharyngeal (NP/OP) samples for SARS-CoV-2 detection and whole blood for serum anti- SARS-CoV-2 NAbs were obtained from each participant at each encounter.

### Sample size

We calculated the required sample size using the standard proportion formula: N = (p·q)/ (ε/ z)2 [17]; where p = expected seroprevalence (2%), q = 100 − p (97.5), z = 1.96 (for 95% CI) and ε = margin of sampling error. Because COVID‑19 was novel at the time, few published studies were available to inform assumptions about expected seroprevalence or the acceptable margin of error for our study setting. Using ε = 2% yields a base sample size of ≈234 at each cross-sectional survey, for a total of 1404. In the end, we enrolled 1,329 (5.3% fewer). This reduced the margin of error to ~±2.05% overall, a negligible loss of precision.

### Eligibility criteria

#### Inclusion criteria.

All individuals identified for recruitment into the investigation, irrespective of age.

Resides within the HDSS

#### Exclusion criteria.

Refusal to give informed consent, or contraindication to venipuncture.

### Laboratory assays

**Sample processing:** NP/OP swabs and EDTA blood were transported on the same day in cool boxes containing ice to the WRAIR-Africa’s Basic Science Lab in Kondele, Kisumu. Nucleic acids were extracted from NP/OP samples using MagMax CORE Nucleic Acid Purification kits (ThermoFisher Scientific, CA USA) according to the manufacturer’s instructions and used for detection of SARS-CoV-2. Serum was separated and then stored at -80 °C until required and thereafter used for detection of anti- SARS-CoV-2 NAbs.

### Detection of SARS-CoV2

TaqPath COVID-19 Combo kit (ThermoFisher Scientific) that targets the ORF 1ab, S and N genes of SARS-CoV-2 and an added bacteriophage (MS2) as an extraction process control was used according to the manufacturer’s instructions. The target sequences are unique to SARS-CoV-2, thus avoiding detection of other coronaviruses. SARS-CoV-2 was considered present if two or more targets (ORF1ab, S, and N genes) were amplified by RT-qPCR. Amplification of only one target was considered inconclusive, and the sample was retested. Lack of amplification of all the targets was reported as negative.

### RBD–ACE2 binding inhibition assay

Surrogate NAbs were measured using the Wantai SARS‑CoV‑2 NAbs ELISA kit (Szabo‑Scandic, China). The assay is a two‑step, solid‑phase competitive ELISA performed according to the manufacturer’s instructions with minor clarifications as follows. Polystyrene microplate wells pre‑coated with recombinant RBD (S‑RBD) were incubated with serum samples (tested in duplicate) to allow any anti‑RBD NAbs to bind S‑RBD. After washing to remove unbound material, HRP‑conjugated to anti S‑RBD was added and plates were incubated to permit binding of the conjugate to any unoccupied S‑RBD sites. Plates were washed and TMB substrate added; the enzymatic reaction was stopped and absorbance measured at 450 nm on a BioTek plate reader (Agilent Technologies Ltd, Santa Clara, CA). NAb concentration was determined from the linear range of the provided lyophilized standards (0.0625, 0.125, 0.25, 0.5 U/mL), run in duplicate on each plate. Samples with values above 0.5 U/mL were diluted with the supplied diluent and re‑assayed. Because the assay is competitive, sample absorbance is inversely proportional to NAb level. Binding inhibition rate (BIR, %) was calculated as: BIR = [(Astandard 0 U/mL − Aspecimen)/ Astandard 0 U/mL] × 100. A BIR ≥ 50% was used as the threshold for positive neutralization.

### Statistical methods

Absorbance values were expressed as the median, mean and interquartile range. Categorical variables like age and gender were expressed as counts and percentages. All statistical analyses were performed using non-parametric tests in GraphPad Prism 9.5.0. Antibody concentrations were reported as log_10_ neutralizing antibody titers. The antibody titers were graphed as geometric mean (GMT) with 95% confidence interval (CI). Unpaired t-test was used to evaluate the mean difference across the cross-sectional surveys, gender and age bands. The probability value of p < 0.05 was considered statistically significant.

## Results

### Study period in relation to Kenya’s COVID-19 outbreak

**Fig 1:**
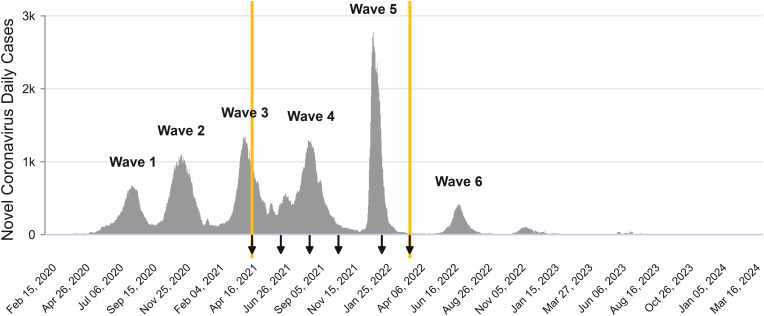
Reported daily new cases in Kenya, 7-day rolling average showing the six distinct waves. https://www.worldometers.info/coronavirus/country/kenya/. The orange lines demarcate the performance period of the study. The arrows indicate timing of the six cross-sectional surveys.

Our study was carried out between April 2021 and March 2022 that coincided with three COVID-19 waves: Alpha (wave 3), Delta (wave 4) and Omicron (wave 5) (Fig 1).

### Demographic characteristics of study participants

A total of 1,329 participants were enrolled during the six cross-sectional study (Table 1). Majority of the study participants were females (69%, 917/1329). The age bands (years) of participants were: < 9 (25%), 10–19 (16%), 20–39 (29%), 40–59 (17%), > 60 (13%).

**Table 1:** Summary of the population characteristics.

Characteristic	Category	Participants, No.	Participants, (%) (N = 1,329)
Gender	*Male*	412	31
	*Female*	917	69
Age	*<9*	334	25
	*10-19*	211	16
	*20-39*	388	29
	*40-59*	225	17
	*≥60*	171	13

### Temporal trends in anti-SARS-CoV-2 NAbs and SARS-CoV-2

**Fig 2:**
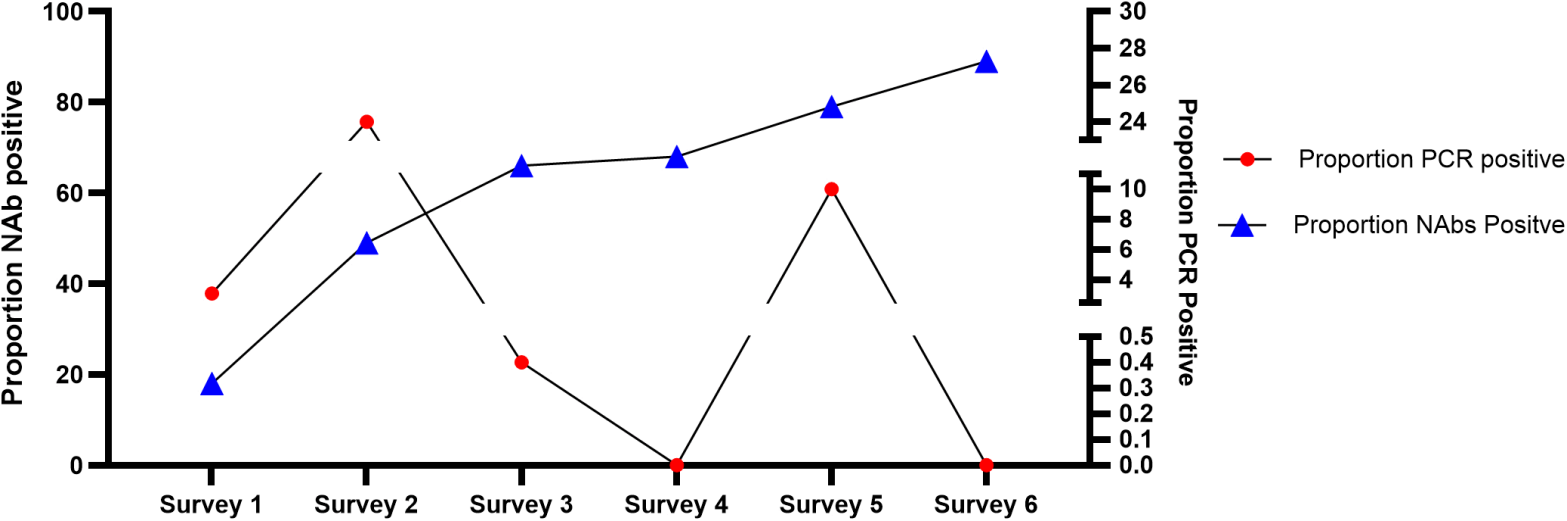
Proportion of participants with neutralizing antibodies and SARS-CoV-2 across the six cross-sectional surveys.

In total and throughout the testing period, out of the 1,329 study participants, 821 (62%) developed anti-SARS-CoV-2 Nabs. A higher proportion of these seropositive individuals, 72% (588/821) were females. As illustrated in Fig 2, Nabs were acquired quickly. At survey one (April 2021), only 14.7% (n = 33/224) of participants had detectable NAbs. Over successive sampling rounds, the proportion of participants with Nabs rose steadily, reaching 88.3% (202/234) by survey six (March 2022). The proportion of participants with RT‑qPCR‑confirmed SARS-CoV-2 infections was only 3.1% (7/224) at study survey one (April 2021), rose to 24.0% (55/229) by survey two (June 2021), then declined to undetectable levels during August 2021 and October 2021 surveys, only to surge to 29.1% (68/234) during the Omicron wave in January 2022, before declining again to zero by March 2022.

**Fig 3:**
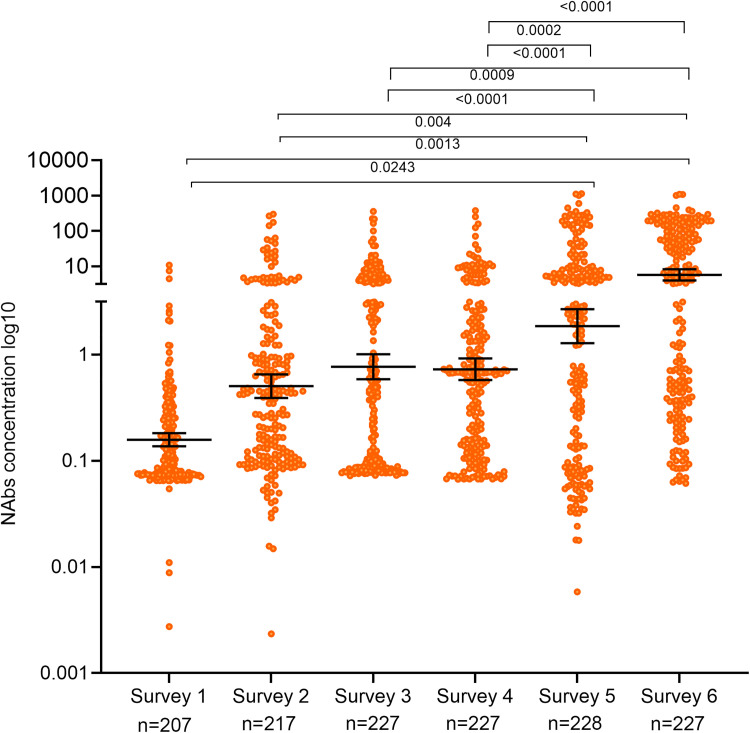
Scatter plot showing the anti-SARS-CoV-2 NAbs levels across the 6 cross-sectional surveys. The mean concentration of NAb steadily increased over time. Center lines represent geometric mean, and whiskers represent the 95% confidence interval. The increase in concentration of anti-SARS-CoV NAbs was significant between survey one and subsequent sampling.

We next measured the concentration of anti-SARS-CoV NAbs for each cross-sectional survey (Fig 3). From cross-referencing the identity of participants, we confirmed that each survey included independent participants. The mean anti-SARS-CoV NAbs concentration rose from 0.42 U/mL (95%, CI, 0.2-0.7) at survey baseline (April 2021), increasing to 1.28 U/mL (95% CI, 0.8-1.9) at survey 2 (June 2021) and 2.64 U/mL (95% CI, 2.0-3.5) at survey 3 (August 2021), dipped to 1.71 U/mL (95% CI, 1.3-2.2) at survey 4, (October 2021), then increased to 4.48U/mL (95% CI, 3.1-6.4) at survey 5 (January 2022) and 9.40U/mL (95% CI, 6.6-13.5) by survey 6 (March 2022). The increase in concentration of anti-SARS-CoV NAbs was significant between survey one and subsequent sampling.

### Females had higher anti-SARS-CoV-2 NAbs than males

**Fig 4:**
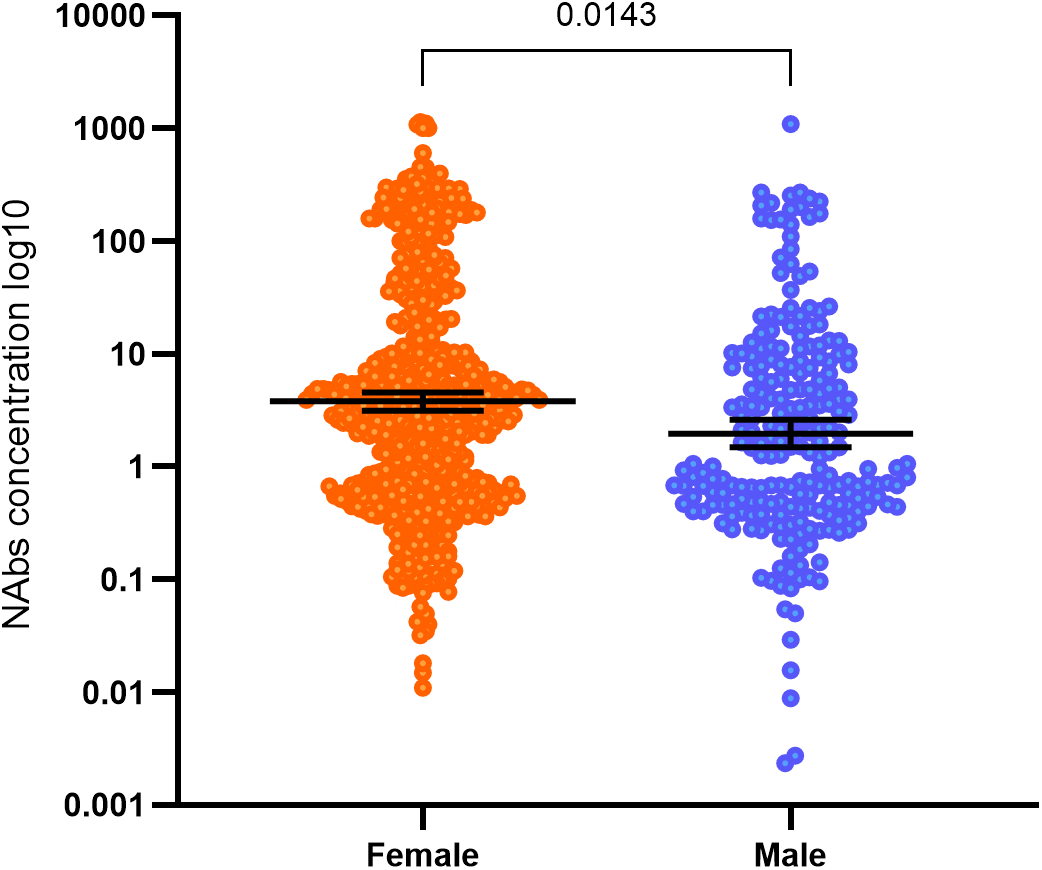
Scatter plot showing anti-SARS-CoV-2 NAbs levels by gender. Overall, NAbs concentration were higher in females than males. Circles represent NAb level per participant. Center lines represent GM and whiskers represent the 95% confidence interval.

**Fig 5:**
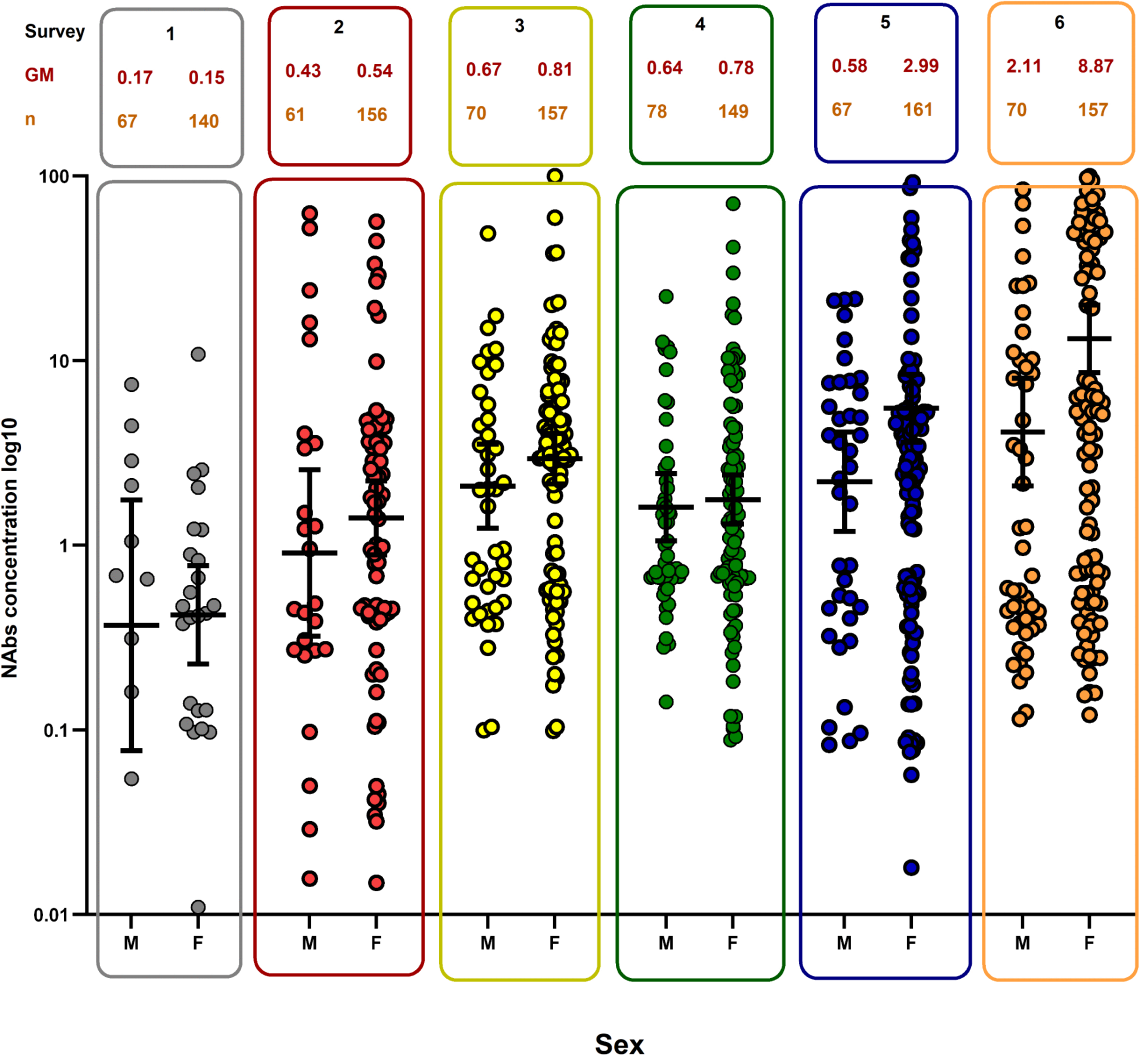
Scatter plot showing anti-SARS-CoV-2 NAbs levels by gender at each cross-sectional survey. Females had higher level of NAbs compared to the male counterparts. Circles represent NAb level per participant. Center lines represent GM and whiskers represent the 95% confidence interval.

The mean NAbs levels in females from all the cross-sectional surveys was higher at 3.77 U/mL (95% CI, 3.1-4.6), compared to males at 1.96 U/mL (95% CI, 1.5-2.6) (p = 0.01432) (Fig 4, panel A). This trend was also observed at each cross-sectional survey (Fig 5).

### Anti-SARS-CoV-2 neutralizing antibody levels by age

**Fig 6:**
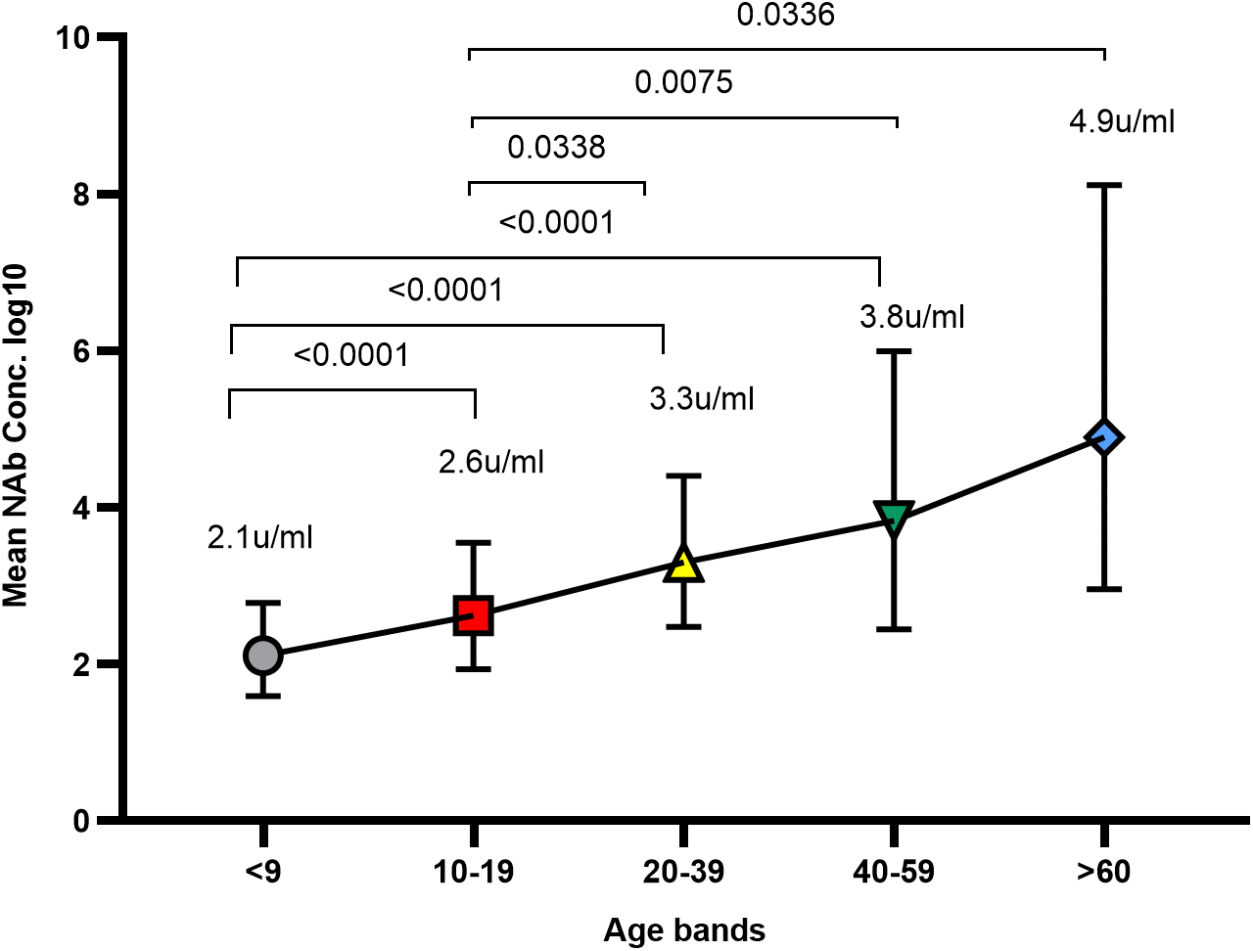
Neutralizing antibody titers based on age. Overall, NAbs concentration increased with age. Titers are graphed as geometric mean titers with 95% Confidence Interval (CI).

**Fig 7:**
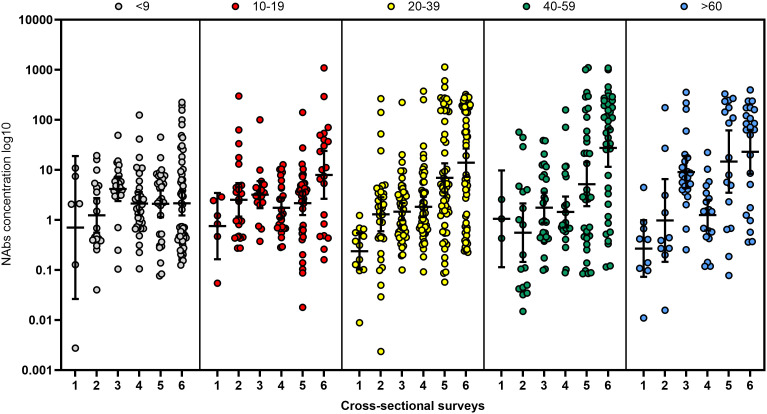
Neutralizing antibody titers based on age at each cross-sectional survey. At each survey, NAbs concentration were higher in the older age categories. Titers are graphed as geometric mean titers with 95% Confidence Interval (CI).

As shown in Fig 6, the mean anti-SARS-CoV-2 NAbs geometric mean titers were higher in older age groups. From 2.1U/mL (95% CI, 1.5-2.8) for <9 years, 2.6U/mL (95% CI, 1.9-3.5) for 10–19 years, 3.3U/mL (95% CI, 2.4-4.4) for 20–39 years, 3.8U/mL (95% CI, 2.4-5.9) for 40 – 59 age band, with a peak observed at age > 60 years at 4.9U/mL (95% CI, 2.9-8.1). This increase was statistically significant overall (Fig 6) and at each survey (Fig 7).

## Discussion

In this year‑long, repeated cross‑sectional study of 1,329 unvaccinated rural participants, we used a sVNT to quantify anti‑SARS‑CoV‑2 Nabs that block RBD–ACE2 interaction and RT‑qPCR to detect active SARS‑CoV‑2 infections, with the goal of characterizing temporal patterns of infection and population‑level NAb acquisition. The studied population was monitored routinely at individual household level for vaccinations, illness, migration, births and deaths. So, apart from two individuals, we are confident that the community was largely unvaccinated, and therefore the observed population‑level NAbs was because of exposure to SARS-CoV-2. Despite COVID‑19 having reached Kenya a year earlier [21] and two national waves having occurred by the time our study started (Fig 1), our April 2021 cross‑sectional survey conducted during the Alpha wave found only 3.1% active infections and 14.7% serologic evidence of prior exposure (Fig 2). Thus, viral penetration in this rural community remained low, likely because those early waves were dominated by B.1 lineages with lower attack rates than later VOCs [22–25]. By survey 2 (June 2021), that was at the tail of the Alpha wave, SARS‑CoV‑2 prevalence and NAb seropositivity had risen to 24% and 40%, respectively, likely driven by Alpha’s increased transmissibility. During surveys 3 and 4 (August and October 2021), coinciding with Delta circulation, NAb prevalence increased to ~60% by survey 3 but plateaued around 60% at survey 4. Infection rates fell to zero only to surge to 29.1% by December 2021–January 2022 (survey 5), at the height of the Omicron wave during which time NAb prevalence reached ~80% (Fig 2). The extensive antigenic change and higher transmissibility amongst the Omicron variants were blamed for producing large waves of infections despite prior population immunity [26–29]. It is tempting to speculate that the observed reduction in RT-qPCR‑confirmed SARS-CoV-2 infections following successful Alpha, Delta and Omicron waves was as a result of cumulative NAbs to different variants. However, the sVNT assesses inhibition of RBD–ACE2 binding rather than direct viral replication in permissive cells. The results should therefore be interpreted as surrogate indicators of neutralizing activity. Nevertheless, sVNT has been shown to correlate strongly with live-virus neutralization assays in multiple published reports [13,30,31].

Similar to the rise in proportion of participants with NAbs, the NAb concentration increased over time except for a dip observed in survey 4 that coincided with the Delta VOC (Fig 3). A study conducted by [32] indicated reduced sensitivity of the Delta variant to NAb, probably due to mutation accumulation in the RBD [33]. The T478K mutation in the RBD is unique to the Delta variants and falls within the epitope region of potent neutralizing antibodies class 1 [34]. This mutation is close to E484K mutation that facilitates antibody escape [35]. Despite the rapid rise in proportion of individuals with Nabs, up to 80% (Fig 2) and 10x increase in NAb concentration (from 0.42 U/mL to 4.48 U/mL) (Fig 3) by survey 5, SARS-CoV-2 infection increased from zero at survey 4 to 29.1% during survey 5 that coincided with the Omicron VOC. The Omicron variant which dominated at this period was reported to have an unusually large numbers of mutations, including more than 30 in the spike protein. The RBD protein which is responsible for interacting with the ACE2 receptor contained 15 of these mutations including K417N and N501Y which are known to contribute to immune escape and higher infectivity [36].

We acknowledge the higher proportion of female participants (69%), likely reflecting greater availability at home during the surveys. Although this may have introduced participation bias, it does not explain the observed high seropositivity rates (72%) or the elevated neutralizing antibody titers (3.77 U/mL (95% CI, 3.1-4.6) compared to males (1.96 U/mL (95% CI, 1.5-2.6) (p = 0.01432) (Figs 4 and 5). This finding is consistent with Zeng et al., [37] who reported higher SARS‑CoV‑2 IgG levels in female COVID‑19 patients during both early and late disease phases, and with Heriyanto et al., [38] who observed significantly higher NAb titers in females at 1, 2 and 3 months post‑vaccination. A proposed explanation for this sex difference is in the location of ACE2 on the X chromosome; females, having two X chromosomes may exhibit different ACE2 expression patterns, which alongside with ACE2’s immunomodulatory roles could influence immune responses and clinical outcomes [25]. Sex‑based differences in immune function and clinical severity favoring females have been reported in multiple studies [23].[39,40]

As shown in Figs 6 and 7, anti-SARS-CoV-2 NAb titers rose progressively with age, consistent with prior work reporting an age-related increase in median NAb levels [26], likely reflecting cumulative exposures. This is in line with a previous study that demonstrated an age-related increase in median NAb titers [26,41], probably because of repeated exposures. In the current study cohort, participants aged > 40 years exhibited the highest level of NAb titers. [42]

In conclusion, this study describes an increase in population level NAbs that is consistent with widespread exposure to SARS-CoV-2 in a rural community in western Kenya that was largely unvaccinated. As a limitation, the study did not collect other covariates that may have influenced transmission, including behavioral changes, adherence to non‑pharmaceutical interventions, and potential cross‑reactive immunity from other coronaviruses [43,44]. Caution is therefore required in interpretation of causality between rising NAb levels and reduced infections rates. In addition, the absence of anti-nucleocapsid antibody data limits differentiation between infection- and vaccine-induced immunity. Despite these limitations, the study provides useful population-level evidence on the temporal dynamics of SARS-CoV-2 infection and antibody responses in a rural African setting.

## Supporting information

S1 Data Statistics for neutralizing antibodies concentration across Surveys 1 – 6 (Fig 3). The table shows the number of observations (n), minimum, maximum, range, arithmetic mean, standard deviation, standard error of the mean, geometric mean, and geometric standard deviation factor, highlighting variability and distribution across survey rounds.
(XLSX)

S2 Data Unpaired two-tailed t test comparing neutralizing antibodies concentration between males and females (Fig 4). Male values were significantly lower than female values (P = 0.0143). Variances differed significantly by F test (F = 2.074, P < 0.0001).
(XLSX)

S3 Data Supplementary data for Fig 5: Summary descriptive statistics by gender (Fig 5). The table shows the number of observations (n), minimum, maximum, and range, arithmetic average; standard deviation (SD), standard error of the mean (SEM), geometric mean and geometric SD factor. The lower and upper 95% CI indicate the confidence interval for the geometric mean.
(XLSX)

S4 Data Supplementary data for Fig 6: Descriptive statistics of neutralizing antibodies concentration by age group (Fig 6). The table shows the number of observations (n), minimum, maximum, and range, arithmetic average; standard deviation (SD), standard error of the mean (SEM), geometric mean and geometric SD factor. The lower and upper 95% CI indicate the confidence interval for the geometric mean.
(XLSX)

S5 Data Supplementary data for Fig 7: Descriptive statistics by age group and survey rounds (Fig 7). The table shows the number of observations (n), minimum, maximum, range, arithmetic mean, standard deviation, standard error of the mean, geometric mean, and geometric standard deviation factor for each subgroup.
(XLSX)

## References

[1] Pang NY-L, Pang AS-R, Chow VT, Wang D-Y. Understanding neutralising antibodies against SARS-CoV-2 and their implications in clinical practice. Mil Med Res. 2021;8(1):47. doi: 10.1186/s40779-021-00342-3 34465396 PMC8405719

[2] Zhu N, Zhang D, Wang W, Li X, Yang B, Song J, et al. A novel coronavirus from patients with pneumonia in China, 2019. N Engl J Med. 2020;382(8):727–33. doi: 10.1056/NEJMoa2001017 31978945 PMC7092803

[3] WHO Director-General’s opening remarks at the media briefing on COVID-19; 2020 [cited 2025 Aug 8]. Available from: https://www.who.int/director-general/speeches/detail/who-director-general-s-opening-remarks-at-the-media-briefing-on-covid-19---11-march-2020

[4] COVID-19 (WHO African region). [cited 2025 Aug 8]. Available from: https://who.maps.arcgis.com/apps/dashboards/0c9b3a8b68d0437a8cf28581e9c063a9

[5] Kenya Confirms First Case of Coronavirus. [cited 2025 Aug 8]. Available from: https://news.scienceafrica.co.ke/kenya-confirms-first-case-of-coronavirus/

[6] COVID-19 Cases, World. [cited 2025 Aug 8]. Available from: https://data.who.int/dashboards/covid19/cases

[7] Naqvi AAT, Fatima K, Mohammad T, Fatima U, Singh IK, Singh A, et al. Insights into SARS-CoV-2 genome, structure, evolution, pathogenesis and therapies: structural genomics approach. Biochim Biophys Acta Mol Basis Dis. 2020;1866(10):165878. doi: 10.1016/j.bbadis.2020.165878 32544429 PMC7293463

[8] Sancilio AE, D’Aquila RT, McNally EM, Velez MP, Ison MG, Demonbreun AR, et al. A surrogate virus neutralization test to quantify antibody-mediated inhibition of SARS-CoV-2 in finger stick dried blood spot samples. Sci Rep. 2021;11(1):15321. doi: 10.1038/s41598-021-94653-z 34321523 PMC8319431

[9] Banga Ndzouboukou J-L, Zhang Y, Fan X-L. Recent developments in SARS-CoV-2 neutralizing antibody detection methods. Curr Med Sci. 2021;41(6):1052–64. doi: 10.1007/s11596-021-2470-7 34935114 PMC8692081

[10] Bewley KR, Coombes NS, Gagnon L, McInroy L, Baker N, Shaik I, et al. Quantification of SARS-CoV-2 neutralizing antibody by wild-type plaque reduction neutralization, microneutralization and pseudotyped virus neutralization assays. Nat Protoc. 2021;16(6):3114–40. doi: 10.1038/s41596-021-00536-y 33893470

[11] Rocha VPC, Quadros HC, Fernandes AMS, Gonçalves LP, Badaró RJ da S, Soares MBP, et al. An overview of the conventional and novel methods employed for SARS-CoV-2 neutralizing antibody measurement. Viruses. 2023;15(7):1504. doi: 10.3390/v15071504 37515190 PMC10383723

[12] Wang JJ, Zhang N, Richardson SA, Wu JV. Rapid lateral flow tests for the detection of SARS-CoV-2 neutralizing antibodies. Expert Rev Mol Diagn. 2021;21(4):363–70. doi: 10.1080/14737159.2021.1913123 33840347 PMC8054491

[13] Tan CW, Chia WN, Qin X, Liu P, Chen MI-C, Tiu C, et al. A SARS-CoV-2 surrogate virus neutralization test based on antibody-mediated blockage of ACE2-spike protein-protein interaction. Nat Biotechnol. 2020;38(9):1073–8. doi: 10.1038/s41587-020-0631-z 32704169

[14] Etyang AO, Adetifa I, Omore R. SARS-CoV-2 seroprevalence in three Kenyan health and demographic surveillance sites, December 2020-May 2021. PLOS Glob Public Health. 2022;2. doi: 10.1371/journal.pgph.0000883 PMC10021917 36962821

[15] Munywoki PK, Bigogo G, Nasimiyu C, Ouma A, Aol G, Oduor CO, et al. Heterogenous transmission and seroprevalence of SARS-CoV-2 in two demographically diverse populations with low vaccination uptake in Kenya, March and June 2021. Gates Open Res. 2023;7:101. doi: 10.12688/gatesopenres.14684.2 37990692 PMC10661969

[16] Uyoga S, Adetifa IMO, Otiende M, Yegon C, Agweyu A, Warimwe GM, et al. Prevalence of SARS-CoV-2 antibodies from a national serosurveillance of Kenyan blood donors, January-March 2021. JAMA. 2021;326(14):1436–8. doi: 10.1001/jama.2021.15265 34473191 PMC8414357

[17] Bergeri I, Whelan MG, Ware H, Subissi L, Nardone A, Lewis HC, et al. Global SARS-CoV-2 seroprevalence from January 2020 to April 2022: a systematic review and meta-analysis of standardized population-based studies. PLoS Med. 2022;19(11):e1004107. doi: 10.1371/journal.pmed.1004107 36355774 PMC9648705

[18] Lewis HC, Ware H, Whelan M, Subissi L, Li Z, Ma X, et al. SARS-CoV-2 infection in Africa: a systematic review and meta-analysis of standardised seroprevalence studies, from January 2020 to December 2021. BMJ Glob Health. 2022;7(8):e008793. doi: 10.1136/bmjgh-2022-008793 35998978 PMC9402450

[19] Mathieu E, Ritchie H, Rodés-Guirao L, et al. Coronavirus (COVID-19) vaccinations. Available from: https://ourworldindata.org/covid-vaccinations

[20] Sifuna P, Oyugi M, Ogutu B, Andagalu B, Otieno A, Owira V, et al. Health & demographic surveillance system profile: the Kombewa health and demographic surveillance system (Kombewa HDSS). Int J Epidemiol. 2014;43(4):1097–104. doi: 10.1093/ije/dyu139 25009309 PMC4258789

[21] Ministry of Health. First case of coronavirus disease confirmed in Kenya; 2020. Available from: https://www.health.go.ke

[22] Davies NG, Abbott S, Barnard RC, Jarvis CI, Kucharski AJ, Munday JD, et al. Estimated transmissibility and impact of SARS-CoV-2 lineage B.1.1.7 in England. Science. 2021;372(6538). doi: 10.1126/science.abg3055 PMC8128288 33658326

[23] Volz E, Mishra S, Chand M, et al. Assessing transmissibility of SARS-CoV-2 lineage B.1.1.7 in England. Nature. 2021;593:266–9. 33767447 10.1038/s41586-021-03470-x

[24] Campbell F, Archer B, Laurenson-Schafer H, Jinnai Y, Konings F, Batra N, et al. Increased transmissibility and global spread of SARS-CoV-2 variants of concern as at June 2021. Eurosurveillance. 2021;26(24):2100509. doi: 10.2807/1560-7917.ES.2021.26.24.2100509 34142653 PMC8212592

[25] Lyngse FP, Mølbak K, Skov RL, Christiansen LE, Mortensen LH, Albertsen M, et al. Increased transmissibility of SARS-CoV-2 lineage B.1.1.7 by age and viral load. Nat Commun. 2021;12(1):7251. doi: 10.1038/s41467-021-27202-x 34903718 PMC8669007

[26] Planas D, Saunders N, Maes P, Guivel-Benhassine F, Planchais C, Buchrieser J, et al. Considerable escape of SARS-CoV-2 Omicron to antibody neutralization. Nature. 2022;602(7898):671–5. doi: 10.1038/s41586-021-04389-z 35016199

[27] Dejnirattisai W, Huo J, Zhou D. SARS-CoV-2 Omicron-B.1.1.529 leads to widespread escape from neutralizing antibody responses. Cell. 2022;185:467-484.e15. 10.1016/j.cell.2021.12.046 PMC8723827 35081335

[28] Cele S, Jackson L, Khoury DS, Khan K, Moyo-Gwete T, Tegally H, et al. Omicron extensively but incompletely escapes Pfizer BNT162b2 neutralization. Nature. 2022;602(7898):654–6. doi: 10.1038/s41586-021-04387-1 35016196 PMC8866126

[29] Pulliam JRC, van Schalkwyk C, Govender N, von Gottberg A, Cohen C, Groome MJ, et al. Increased risk of SARS-CoV-2 reinfection associated with emergence of Omicron in South Africa. Science. 2022;376(6593). doi: 10.1126/science.abn4947 PMC8995029 35289632

[30] Valcourt EJ, Manguiat K, Robinson A, Chen JC-Y, Dimitrova K, Philipson C, et al. Evaluation of a commercially-available surrogate virus neutralization test for severe acute respiratory syndrome coronavirus-2 (SARS-CoV-2). Diagn Microbiol Infect Dis. 2021;99(4):115294. doi: 10.1016/j.diagmicrobio.2020.115294 33387896 PMC7758721

[31] Perera RA, Mok CK, Lv H, et al. Serological assays for severe acute respiratory syndrome coronavirus 2 (SARS-CoV-2). Eurosurveillance. 2020. doi: 10.2807/1560-7917.ES.2020.25.16.2000421 PMC7189648 32347204

[32] Planas D, Veyer D, Baidaliuk A, Staropoli I, Guivel-Benhassine F, Rajah MM, et al. Reduced sensitivity of SARS-CoV-2 variant Delta to antibody neutralization. Nature. 2021;596(7871):276–80. doi: 10.1038/s41586-021-03777-9 34237773

[33] Starr TN, Greaney AJ, Addetia A, et al. Prospective mapping of viral mutations that escape antibodies used to treat COVID-19; 2021. Available from: https://jbloomlab.github.io/SARS-CoV-2-RBD_MAP_clinical_Abs/10.1126/science.abf9302 PMC7963219 33495308

[34] Barnes CO, Jette CA, Abernathy ME, Dam K-MA, Esswein SR, Gristick HB, et al. SARS-CoV-2 neutralizing antibody structures inform therapeutic strategies. Nature. 2020;588(7839):682–7. doi: 10.1038/s41586-020-2852-1 33045718 PMC8092461

[35] Plante JA, Mitchell BM, Plante KS, Debbink K, Weaver SC, Menachery VD. The variant gambit: COVID-19’s next move. Cell Host Microbe. 2021;29(4):508–15. doi: 10.1016/j.chom.2021.02.020 33789086 PMC7919536

[36] Cao Y, Wang J, Jian F, Xiao T, Song W, Yisimayi A, et al. Omicron escapes the majority of existing SARS-CoV-2 neutralizing antibodies. Nature. 2022;602(7898):657–63. doi: 10.1038/s41586-021-04385-3 35016194 PMC8866119

[37] Zeng F, Dai C, Cai P, Wang J, Xu L, Li J, et al. A comparison study of SARS-CoV-2 IgG antibody between male and female COVID-19 patients: a possible reason underlying different outcome between sex. J Med Virol. 2020;92(10):2050–4. doi: 10.1002/jmv.25989 32383183 PMC7267228

[38] Heriyanto RS, Kurniawan A, Wijovi F. The role of COVID‐19 survivor status and gender towards neutralizing antibody titers 1, 2, 3 months after Sinovac vaccine administration on clinical‐year medical students in Indonesia: role of COVID‐19 survivor status and gender towards neutralizing antibody titers after Sinovac vaccine administration. Int J Infect Dis. 2021;113:336–8. 34653654 10.1016/j.ijid.2021.10.009 PMC8507582

[39] Heriyanto RS, Kurniawan A, Wijovi F, et al. The role of COVID‐19 survivor status and gender towards neutralizing antibody titers 1, 2, 3 months after Sinovac vaccine administration on clinical‐year medical students in Indonesia: role of COVID‐19 survivor status and gender towards neutralizing antibody titers after Sinovac vaccine administration. Int J Infect Dis. 2021;113:336–8. 34653654 10.1016/j.ijid.2021.10.009 PMC8507582

[40] Ciarambino T, Para O, Giordano M. Immune system and COVID-19 by sex differences and age. Womens Health (Lond). 2021;17:17455065211022262. doi: 10.1177/17455065211022262 34096383 PMC8188967

[41] Kim Y, Bae J-Y, Kwon K, Chang H-H, Lee WK, Park H, et al. Kinetics of neutralizing antibodies against SARS-CoV-2 infection according to sex, age, and disease severity. Sci Rep. 2022;12(1):13491. doi: 10.1038/s41598-022-17605-1 35931794 PMC9356129

[42] Statsenko Y, Al Zahmi F, Habuza T, et al. Impact of age and sex on COVID-19 severity assessed from radiologic and clinical findings. Front Cell Infect Microbiol. 2022;11. doi: 10.3389/fcimb.2021.777070 PMC8913498 35282595

[43] Ng KW, Faulkner N, Cornish GH, et al. Preexisting and de novo humoral immunity to SARS-CoV-2 in humans. Available from: https://www.science.org 10.1126/science.abe1107 PMC7857411 33159009

[44] Sagar M, Reifler K, Rossi M. Recent endemic coronavirus infection is associated with less-severe COVID-19. J Clin Investig. 2021;131. doi: 10.1172/JCI143380 PMC7773342 32997649

