# Supplementary material for: SARS-CoV-2 and neutralizing antibody dynamics at a remote village in Kenya before widespread COVID-19 vaccine rollout
Source: PLOS Glob Public Health. 2026 Jul 24;6(7):e0006706. doi: 10.1371/journal.pgph.0006706 (PMC13399503; doi:10.1371/journal.pgph.0006706)

PLOS Global Public Health

SARS-CoV-2 and neutralizing antibody dynamics at a remote village in Kenya before COVID-19 vaccine rollout

--Manuscript Draft--

Manuscript Number:	PGPH-D-26-00167
Article Type:	Research Article
Full Title:	SARS-CoV-2 and neutralizing antibody dynamics at a remote village in Kenya before COVID-19 vaccine rollout
Short Title:	SARS-CoV-2 and neutralizing antibody dynamics before COVID-19 vaccine rollout
Corresponding Author:	John Waitumbi<br>KEMRI: Kenya Medical Research Institute<br>Kisumu, N/A KENYA
Order of Authors:	Esther Omuseni
	Josphat Nyataya
	Peter Sifuna
	George Awinda
	Allan Lemtudo
	Clement Masakhwe
	Beth Mutai
	Geoffrey Maina, PhD
	Simon Kariuki, PhD
	John Waitumbi
Keywords:	SARS-CoV-2; Neutralizing Antibodies; COVID-19, Herd Immunity, COVID-19 Vaccine
Abstract:	Objective: The study evaluated SARS-CoV-2 transmission and the buildup of herd immunity at a rural population in western Kenya that had not received COVID-19 vaccine.<br>Design: Six cross-sectional surveys, two months apart were conducted between April 2021 and April 2022, during which time, the Alpha, Delta and Omicron waves dominated. At each survey, participants provided naso-pharyngeal samples for SARS-CoV-2 and serum for neutralizing anti-SARS-CoV-2 antibodies (NAbs).<br>Results: 69% of the participants recruited (n=1329) were females. SARS-CoV-2 increased from 3.4% at survey one to 24.6% by month two, and thereafter decreased to zero, only to rise to 10% during the Omicron wave. Participants with NAbs increased from 18% at survey one to 89% by end of the study. The quantity of NAbs rose from a geometric mean 0.42 U/mL at survey one to 9.4 U/mL by survey six. Females had higher NAbs (3.73U/mL, 95% CI), compared to 1.98U/mL, 95% CI (p=0.0146) males. NAbs were higher in older age bands: mean of 2.1U/mL, 2.6U/mL, 3.4U/mL, 3.8U/mL and 4.9U/mL for ages <9 Years, 10-19 years, 20-39 years, 40-59 years and >60 years respectively.<br>Conclusions: The buildup of herd immunity was a result of exposure to COVID-19 and not due to vaccine.
Additional Information:	
Question	Response
Financial Disclosure<br><br>Enter a financial disclosure statement that	The authors declare no financial interest

Powered by Editorial Manager® and ProduXion Manager® from Aries Systems Corporation

describes the sources of funding for the work included in this submission and the role the funder(s) played. This includes grants and any commercial funding of the work or authors.<br><br>This statement will be typeset if the manuscript is accepted for publication.<br><br>*Please review the submission guidelines and the instructions link below for detailed requirements and guidance.*	
**Competing Interests**<br><br>On behalf of all authors, disclose any competing interests that could be perceived to bias this work.<br><br>This statement will be typeset if the manuscript is accepted for publication.<br><br>*Please review the instructions link below and PLOS Global Public Health's competing interests policy to determine what information must be disclosed at submission.*	The authors declare no competing interest
**Data Availability**<br><br>Before publication, Authors are required to make fully available and without restriction all data underlying their findings. Please see our PLOS Data Policy page for detailed information on this policy.<br><br>A **Data Availability Statement**, detailing where the data can be accessed, is required at first submission. Insert your Data Availability Statement in the box below.<br><br>Please see the data reporting section of	All the data are available in the manuscript

our submission guidelines for instructions on what you need to include in your Data Availability Statement.<br><br>This statement will be typeset if the manuscript is accepted for publication.<br><br>*PLOS allows rare exemptions to address legal and ethical concerns. If you have legal or ethical restrictions, please use the box below to detail these in full sentences for the Journal team to consider.*	

Manuscript

Click here to access/download;Manuscript;Omuseni et al_Manuscript_25Jan2026.docx

SARS-CoV-2 and neutralizing antibody dynamics at a remote village in Kenya before COVID-19 vaccine rollout

Esther Omuseni[1], Josphat Nyataya[1], Peter Sifuna[1], George Awinda[1], Allan Lemtudo[1], Clement Masakhwe[1], Beth Mutai[1], Geoffrey Maina[2], Simon Kariuki[3], John Waitumbi[1]*

[1]Kenya Medical Research Institute/Walter Reed Army Institute of Research-Africa, Basic Science Laboratory, Kisumu, Kenya.

[2]Kenya Medical Research Institute, Center Biotechnology Research and Development, Nairobi, Kenya

[3]Kenya Medical Research Institute, Center for Global Health Research, Kisumu, Kenya

*Correspondence: john.waitumbi@usamru-k.org, +254733616548

ABSTRACT

The study evaluated SARS-CoV-2 transmission and the buildup of herd immunity at a rural population in western Kenya that had not received COVID-19 vaccine. Six cross-sectional surveys, two months apart were conducted between April 2021 and April 2022, during which time, the Alpha, Delta and Omicron waves dominated. At each survey, participants provided naso-pharyngeal samples for SARS-CoV-2 and serum for neutralizing anti-SARS-CoV-2 antibodies (NAbs). 69% of the participants recruited (n=1329) were females. SARS-CoV-2 increased from 3.4% at survey one to 24.6% by month two, and thereafter decreased to zero, only to rise to 10% during the Omicron wave. Participants with NAbs increased from 18% at survey one to 89% by end of the study. The quantity of NAbs rose from a geometric mean 0.42 U/mL at survey one to 9.4 U/mL by survey six. Females had higher NAbs (3.73U/mL, 95% CI), compared to 1.98U/mL, 95% CI (p=0.0146) males. NAbs were higher in older age bands: mean of 2.1U/mL, 2.6U/mL, 3.4U/mL, 3.8U/mL and 4.9U/mL for ages <9 Years, 10-19 years, 20-39 years, 40-59 years and >60 years respectively. The buildup of herd immunity was a result of exposure to COVID-19 and not due to vaccine.

Keywords: SARS-CoV-2, Neutralizing Antibodies, ELISA

INTRODUCTION

The coronavirus disease 2019 (COVID-19) pandemic was caused by an emergent beta-coronavirus (SARS-COV-2) [1] that first appeared in Wuhan, China in December 2019 [2]. The virus rapidly spread globally and on 11 March 2020, the World Health Organization declared the disease a global pandemic [3]. By the time COVID-19 was declared no longer “a public health emergency of international concern” in March 2023, it had infected about 765 million people globally and killed nearly 7 million died of virus. Of these deaths, only 174,211 were reported from Africa [4]. In Kenya, the first case was traced to a Kenyan citizen who arrived in Nairobi from the United States on 5th March 2020 [5] . By March 2023, there had been 342,967 confirmed cases of COVID-19 and 5,689 deaths [6].

Like other coronaviruses, SARS-CoV-2 contains four structural proteins that include spike (S), envelope (E), membrane (M) and nucleocapsid (N). The virus uses the spike protein to bind to the host cell-surface receptor through the Receptor Binding Domain (RBD) protein in the S1 subunit, followed by fusion of the S2 subunit to the cell membrane during host cell entry [7]. In addition to playing a role in determining the viral host range, and infectivity, the S protein is a critical target for inducing SARS-CoV-2 specific NAbs that correlate to immunity [1]. NAbs are crucial for viral clearance and protection against SARS-CoV-2 by interfering with virion binding to RBD. Measurement of Nab to RBD provides information on the magnitude of immune response resulting from disease and vaccination [8].

Neutralizing activity can be determined in multiple ways, including plaque reduction neutralization test (PRNT) [9]using wild-type virus, pseudovirus (pVNA) [10] and ELISA-based antibody binding assays using surrogate virus [11]. Although the PRNT is the gold standard for measurement of neutralizing activity, the assay is labor-Intensive, time-consuming and can have

high variability depending on cell line, virus strain, operator competence and requires BSL3. pVNA has similar drawbacks as the PRNT. The ELISA-based antibody binding assays are easier, but measure binding, not neutralization. Several ELISAs tests have been developed for the detection and quantification of SARS-CoV-2 NAbs [9] that measure binding to recombinant RBD protein or angiotensin 2 converting enzyme (ACE2) as the coating Ag and horseradish peroxidase (HRP) labeled ACE2 or RBD labeled HRP to produce a detection signal [9]. Neutralizing antibodies can be raised only against S protein. This is because SARS-CoV-2 invades its host via interaction of its S protein with ACE-2 protein on the surface of host cells [12]. Surrogate Virus Neutralization Test (sVNT) detects neutralizing antibodies without the need for any live virus or cells and can be completed in 1-2 hours in a BSL2 laboratory [13]. In this study, we report on the SARS-CoV-2 and neutralizing antibody dynamics to RBD protein using a surrogate virus neutralization test in participants enrolled in a cross-sectional study at the WRAIR-Africa's Health and Demographic Surveillance Systems (HDSS) site in Kombewa, Western Kenya.

MATERIALS AND METHODS

Study Site and Population

The study was conducted at the WRAIR-Africa's HDSS site in Kombewa, western Kenya between April 2021 and March 2022. The study site has a population of about 160,000 inhabitants who live in about 30,000 households. The study was open to all age groups and gender living within the HDSS households. Participants were requested to provide vaccination information. By the end of the study, only two participants were confirmed to have received COVID-19 vaccine.

Ethical Approval

Study participants were consented to take part in the study and ethical approval was provided by the Kenya Medical Research Institute (KEMRI) Scientific and Ethical Research Unit (SERU) (KEMRI/SERU #4033) and the Walter Reed Army Institute of Research (WRAIR) Human Subject Protection Branch (HSPB) (WRAIR/HSPB #2813). Informed written consent was obtained from all participants and documented by witnessed signature. Parents or legal guardians provided consent on behalf of the minors. In addition to consent provided by parents or legal guardians, emancipated minors (ages 7 and 17 years) provided wriiten assent to indicate that they agreed to take part in the study.

Study Design and Sampling

This was a prospective study conducted through six cross-sectional surveys spaced two months apart over a period of twelve months. The HDSS data manager selected the households with appropriate age band categories and invited the members to participate at each cross-sectional encounter. Prior to participation, informed consent/assent was obtained from the participants. Consenting was done in English or the local language if desired i.e. Kiswahili or Luo. Nasopharyngeal/oral-pharyngeal (NP/OP) samples for SARS-CoV-2 detection and whole blood for anti- SARS-CoV-2 NAbs were obtained from each participant at each encounter. Study participants with SARS-CoV-2 or anti- SARS-CoV-2 NAbs were enrolled for longitudinal study whence NP/OP were taken every 2 weeks and blood monthly.

Laboratory Assays

Sample processing: NP/OP swabs and EDTA blood were transported on the same day in cool boxes containing ice to the WRAIR-Africa’s Basic Science Lab in Kondele, Kisumu. NP/OP samples were processed immediately for nucleic acids extraction using MagMax CORE Nucleic

Acid Purification kits (ThermoFisher Scientific, CA USA) according to the manufacturer's instructions and used for detection of SARS-CoV-2. Plasma was separated and then stored at -80 °C until required and thereafter used for detection of anti- SARS-CoV-2 NAbs.

Detection of SARS-CoV2

TaqPath COVID-19 Combo kit (ThermoFisher Scientific) that targets the ORF 1ab, S and N genes of SARS-CoV-2 and an added bacteriophage (MS2) as an extraction process control was used according to the manufacturer's instructions. The target sequences are unique to SARS-CoV-2, thus avoiding detection of other coronaviruses. Presence of SARS-CoV-2 was reported if two or more SARS-CoV-2 targets by RT-qPCR were amplified. Amplification of only one target was considered inconclusive, and the sample was retested. The lack of amplification of all the targets was reported as negative.

Neutralizing antibodies to SARS-CoV-2 RBD protein by ELISA.

Serum samples were tested with a surrogate viral neutralizing assay kit (Wantai SARS-CoV-2 NAbs ELISA (SZABO SCANDIC). The test employs solid phase, competitive ELISA in a two-step procedure. Briefly, serum samples are added to the polystyrene microwell strips pre-coated with recombinant RBD protein, hence forth referred to as solid phase pre-coated RBD (S-RBD). During the first incubation step, SARS-CoV-2 specific neutralizing antibodies, if present, binds to the S-RBD. The wells are then washed to remove unbound proteins. In the second incubation, S-RBD antibodies conjugated to horseradish peroxidase (HRP-conjugate) are added to bind any free S-RBD from the previous step. Lastly, a substrate is added that results in color change due to enzyme substrate reaction between the conjugated HRP secondary antibodies and the substrate. The intensity of color change was measured on BioTek plate reader (Agilent Technologies Ltd,

Santa Clara CA) at 450nm. The concentration of NAbs in the test sample is inversely correlated to the concentration of HRP-conjugated S-RBD antibodies. The concentration of NAbs was extrapolated from linear range of the standard curve that was drawn from the standard (0.0625 U/mL, 0.125 U/mL, 0.25 U/mL and 0.5 U/mL), supplied in lyophilized form by manufacturer and tested in duplicates alongside test samples. Samples whose concentration was higher than 0.5U/mL were diluted using the provided standard diluent and re-tested. To determine how effective the host neutralizing antibodies prevented the labelled secondary antibodies from binding to the S-RBD, the binding inhibition rate (BIR) was calculated as BIR = (A value of Standard 0 U/mL – A value of specimen) * 100%. A BIR $\geq$ 50% was used as the threshold.

Statistical methods

Absorbance values were expressed as the median, mean and interquartile range. Categorical variables like age and gender were expressed as counts and percentages. All statistical analyses were performed using non-parametric tests in GraphPad Prism 9.5.0. Antibody concentrations were reported as $\log_{10}$ neutralizing antibody titers. The antibody titers were graphed as geometric mean (GMT) with 95% confidence interval (CI). Unpaired t-test was used to evaluate the mean difference across the cross-sectional surveys, gender and age bands. The probability value of $p < 0.05$ was considered statistically significant.

RESULTS

Study period in relation to Kenya's COVID-19 outbreak

Our study was carried out between April 2021 and March 2022 that coincided with three COVID-19 waves: Alpha, Delta and Omicron (Figure 1).

Figure 1: Reported daily new cases in Kenya, 7-day rolling average showing the six distinct waves. https://www.worldometers.info/coronavirus/country/kenya/. The orange lines demarcate the performance period of the study.

Demographic characteristics of study participants

A total of 1,329 participants were enrolled during the six cross-sectional study (Table1). Majority of the study participants were females (67.8%). The age bands (years) of participants were: <9 (26.9%), 10-19 (15.4%), 20-39 (28.4%), 40-59 (16.6%), >60 (12.4%).

Table 1: Summary of the population characteristics

Characteristic	Category	Participants, No.	Participants, (%) (N = 1,385)
**Gender**	*Male*	439	31.7
	Female	939	67.8
**Age**	*<9*	373	26.9
	10-19	213	15.4
	20-39	393	28.4
	40-59	230	16.6
	≥60	172	12.4

Temporal trends in anti-SARS-CoV-2 NAbs and SARS-CoV-2

In total and throughout the testing period, out of the 1,385 study participants, 821 (62%) developed anti-SARS-CoV-2 NAbs. A higher proportion of these (67.8%) were females. As shown in Figure 2, the acquisition of NAbs was rapid. At study start (survey one), out of the 224 participants recruited, 14.7% (n=33) were found to have anti-SARS-CoV-2 NAbs, that increased steadily to

88.3% (202/234) by survey 6. SARS-CoV-2 positivity increased markedly from 3.1% (7/224) at survey one to 24.0% (55/229) by second survey and thereafter decreased to zero and rose again to 29.1% (68/234) during the Omicron wave at survey 5 then decreased to 0% by end of the survey 6.

Figure 2: Proportion of participants with neutralizing antibodies and SARS-CoV-2 across the six cross-sectional surveys. Wave 3 was dominated by Alpha variants, 4 by Delta and 5 by Omicron.

We next measured the concentration of anti-SARS-CoV NAbs (Figure 3). The mean concentration increased steadily from 0.42U/mL (95%, CI, 0.2-0.7) at survey one to 1.28U/mL (95% CI, 0.8-1.9) at survey two, 2.64U/mL (95% CI, 2.0-3.5) at survey 3, 1.71U/mL (95% CI, 1.3-2.2) at survey four, 4.48U/mL (95% CI, 3.1-6.4) and 9.40U/mL (95% CI, 6.6-13.5) at survey six. A slight decline was recorded at survey four. The increase in concentration of anti-SARS-CoV NAbs was significant between survey one and subsequent sampling.

Figure 3: Scatter plot showing the anti-SARS-CoV-2 NAbs levels across the 6 cross-sectional surveys. The mean concentration of NAb steadily increased over time. Center lines represent geometric mean, and whiskers represent the 95% confidence interval. The increase in concentration of anti-SARS-CoV NAbs was significant between survey one and subsequent sampling.

Females had higher anti-SARS-CoV-2 NAbs than males

The mean NAbs levels in females was higher at 3.77U/mL (95% CI, 3.1-4.6), compared to males at 1.96U/mL (95% CI, 1.5-2.6) ($p=0.01432$) (Figure 4, panel A). This was true even at each survey (Figure 4, panel B)

Figure 4: Scatter plot showing anti-SARS-CoV-2 NAbs levels by gender. Overall, NAbs concentration (Panel **A)** and by cross-sectional surveys (Panel **B)** were higher in females than males. Circles represent NAb level per participant. Center lines represent GM and whiskers represent the 95% confidence interval.

Anti-SARS-CoV-2 neutralizing antibody levels by age

As shown in Figure 5, the mean anti-SARS-CoV-2 NAbs were higher in older age groups. From 2.1U/mL (95% CI, 1.5-2.8) for <9 years, 2.6U/mL (95% CI, 1.9-3.5) for 10-19 years, 3.3U/mL (95% CI, 2.4-4.4) for 20-39 years, 3.8U/mL (95% CI, 2.4-5.9) for 40 – 59 age band, with a peak observed at age >60 years at 4.9U/mL (95% CI, 2.9-8.1). This increase was statistically significant overall (Figure 5, pane[illegible] n = 588 [illegible] t each su[illegible] n = 233 [illegible] re 5, panel B).

Figure 5: Neutralizing antibody titers based on age. Overall NAbs concentration (Panel **A)** and by cross-sectional surveys Panel **B)**. Titers are graphed as geometric mean titers (GMT) with 95% Confidence interval (CI).

DISCUSSION

In this study, we evaluated anti- SARS-CoV-2 Nab and SARS-CoV-2 in 1,385 rural participants who took part in a year-long cross-sectional study to understand the temporal acquisition of the virus and buildup of herd immunity in a population that was unlikely to have received COVID-19 vaccination. At the beginning of the study, SARS-CoV-2 infection rates were low (3.1%) and only 14.7% of the study participants had an immunological experience of COVID-19 (Figure 2). Although by then Kenya had gone through the first two waves of COVID-19 (Figure 1), the virus penetration to the studied community was low because these first waves were dominated by B.1 lineages that had lower attack rate compared to subsequent variants of concern (Nasimiyu et al

2022). Immunological experience to COVID-19 dramatically increased, reaching 43.2% two months into the study (Figure 2) and reached 88.3% within one year, indicating the infectiousness of SARS-CoV-2 lineages associated with VOC. Equally, the SARS-CoV-2 rose to 24.0% by second survey (Figure 2) that coincided with the SARS-CoV-2 wave 3 that was caused by Alpha VOC Kimita et al., 2022). Subsequently, as the proportion of individuals with Nab increased to 60% by survey 3 (end of Alpha wave), infection rates had dipped to zero during the Delta wave. Clearly, the rising herd immunity protected the community from the deleterious effect of Delta VOC. SARS-CoV-2 infection rate in the study population thereafter rose to 29.1% during wave 5 that was caused by Omicron VOC (Figure 2).

Similar to the rise in proportion of participants with NAbs, the Nab concentration increased over time except for a dip observed in survey 4 that coincided with the Delta VOC (Figure 3). A study conducted by [14] indicated reduced sensitivity of the Delta variant to NAb, probably due to mutation accumulation in the RBD [15]. The T478K mutation in the RBD is unique to the Delta variants and falls within the epitope region of potent neutralizing antibodies class 1 [16]. This mutation is close to E484K mutation that facilitates antibody escape [17]. Despite the increase in the rapid rise in proportion (Figure 2) and concentration (Figure 3) of NAbs, SARS-CoV-2 infection rose to 29.1% during survey 5 that coincided with the Omicron VOC. The Omicron variant which dominated at this period was reported to have an unusually large number of mutations, including more than 30 in the spike protein. The RBD protein which is responsible for interacting with the ACE2 receptor contained 15 of these mutations including K417N and N501Y which are known to contribute to immune escape and higher infectivity [18].

Female participants had significantly higher anti-SARS-CoV-2 NAb titers than the male counterparts (Figure 4). This is in line with a study carried out by [19] that compared SARS-CoV-

2 IgG antibody levels between male and female COVID-19 patients. In that study, more female patients had a relatively higher levels of IgG antibody after SARS-CoV-2 in early and late phases of COVID-19. Similar results were reported in another study that evaluated whether NAbs titers influenced COVID-19 survivor status 1,2,3 months post vaccination. In that study, female participants had significantly higher NAb titres in all the three months after vaccination [20]. The proposed explanation for this gender disaparity hinges on fact that the ACE2 that represents the primary route of SARS-CoV-2 entry in the human host is located on the X chromosome, and female having two X chromosomes may have higher expression levels of this enzyme. The enzyme also exerts several immunomodulating effects that may explain less severe clinical outcomes in females [21]. This gender disparity that favors females in clinical outcomes of COVID-19 has been confirmed in mutiple studies [19].

As shown in Figure 5, there was a steady increase in anti-SARS-CoV-2 NAb titers with age. This is in line with a previous study that demonstrated an age-related increase in median NAb titers [22]. Based on our data, participants of 40 years and above had the highest level of NAbs titers. A study carried out by [23] on the impact of age on COVID-19 severity reported a worsening trend of SARS-CoV-2 infection with age.

In conclusion, this study catalogues the temporal buildup of disease mediated herd immunity characterized by sequential reduction in proportion of individuals with SARS-CoV-2 and a steady increase in proportion and quantity of anti-SARS-CoV-2 NAbs. Age and gender influenced the level of anti-SARS-CoV-2 titers. As a limitation, we note that we should have included anti-nucleocapsid (N) antibody data that would have distinguished disease from vaccine induced antibody response.

ACKNOWLEDGEMENT

Funding: This work was funded by the Kenya Medical Research Institute (Internal Grants) to JW and also from Armed Forces Health Surveillance Division (AFHSD) and its Global Emerging Infections Surveillance and Research Branch (ProMIS P0095_21_KY; P0091_23_KY and P0089_24_KY) to JW. The funders had no role in data collection and analysis, decision to publish, or preparation of the manuscript.

Disclaimer: Material has been reviewed by the Walter Reed Army Institute of Research. There is no objection to its publication. The opinions or assertions contained herein are the private views of the author, and they are not to be construed as official, or as reflecting true views of the Department of the Army or the Department of Defense. The investigators have adhered to the policies for protection of human subjects as prescribed in AR 70–25.

REFERENCES

[1] Pang NYL, Pang ASR, Chow VT, et al. Understanding neutralising antibodies against SARS-CoV-2 and their implications in clinical practice. *Military Medical Research*; 8. Epub ahead of print 1 December 2021. DOI: 10.1186/s40779-021-00342-3.

[2] Zhu N, Zhang D, Wang W, et al. A Novel Coronavirus from Patients with Pneumonia in China, 2019. *New England Journal of Medicine* 2020; 382: 727–733.

[3] WHO Director-General's opening remarks at the media briefing on COVID-19 - 11 March 2020, https://www.who.int/director-general/speeches/detail/who-director-general-s-opening-remarks-at-the-media-briefing-on-covid-19---11-march-2020 (accessed 8 August 2025).

[4] COVID-19 (WHO African region), https://who.maps.arcgis.com/apps/dashboards/0c9b3a8b68d0437a8cf28581e9c063a9 (accessed 8 August 2025).

[5] Kenya Confirms First Case of Coronavirus, https://news.scienceafrica.co.ke/kenya-confirms-first-case-of-coronavirus/ (accessed 8 August 2025).

[6] COVID-19 Cases, World, https://data.who.int/dashboards/covid19/cases (accessed 8 August 2025).

[7] Naqvi AAT, Fatima K, Mohammad T, et al. Insights into SARS-CoV-2 genome, structure, evolution, pathogenesis and therapies: Structural genomics approach.

Biochimica et Biophysica Acta - Molecular Basis of Disease; 1866. Epub ahead of print 1 October 2020. DOI: 10.1016/j.bbadis.2020.165878.

[8] Sancilio AE, D'Aquila RT, McNally EM, et al. A surrogate virus neutralization test to quantify antibody-mediated inhibition of SARS-CoV-2 in finger stick dried blood spot samples. *Sci Rep* 2021; 11: 1–6.

[9] Banga Ndzouboukou JL, Zhang Y di, Fan X lin. Recent Developments in SARS-CoV-2 Neutralizing Antibody Detection Methods. *Curr Med Sci* 2021; 41: 1052–1064.

[10] Bewley KR, Coombes NS, Gagnon L, et al. Quantification of SARS-CoV-2 neutralizing antibody by wild-type plaque reduction neutralization, microneutralization and pseudotyped virus neutralization assays. *Nature Protocols* 2021; 16: 3114–3140.

[11] Rocha VPC, Quadros HC, Fernandes AMS, et al. An Overview of the Conventional and Novel Methods Employed for SARS-CoV-2 Neutralizing Antibody Measurement. *Viruses*; 15. Epub ahead of print 1 July 2023. DOI: 10.3390/v15071504.

[12] Wang JJ, Zhang N, Richardson SA, et al. Rapid lateral flow tests for the detection of SARS-CoV-2 neutralizing antibodies. *Expert Review of Molecular Diagnostics* 2021; 21: 363–370.

[13] Tan CW, Chia WN, Qin X, et al. A SARS-CoV-2 surrogate virus neutralization test based on antibody-mediated blockage of ACE2–spike protein–protein interaction. *Nat Biotechnol* 2020; 38: 1073–1078.

[14] Planas D, Veyer D, Baidaliuk A, et al. Reduced sensitivity of SARS-CoV-2 variant Delta to antibody neutralization. *Nature* 2021; 596: 276–280.

[15] Starr TN, Greaney AJ, Addetia A, et al. *Prospective mapping of viral mutations that escape antibodies used to treat COVID-19*, https://jbloomlab.github.io/SARS-CoV-2-RBD_MAP_clinical_Abs/ (2021).

[16] Barnes CO, Jette CA, Abernathy ME, et al. SARS-CoV-2 neutralizing antibody structures inform therapeutic strategies. *Nature* 2020; 588: 682–687.

[17] Plante JA, Mitchell BM, Plante KS, et al. The variant gambit: COVID-19's next move. *Cell Host and Microbe* 2021; 29: 508–515.

[18] Cao Y, Wang J, Jian F, et al. Omicron escapes the majority of existing SARS-CoV-2 neutralizing antibodies. *Nature* 2022; 602: 657–663.

[19] Zeng F, Dai C, Cai P, et al. A comparison study of SARS-CoV-2 IgG antibody between male and female COVID-19 patients: A possible reason underlying different outcome between sex. *J Med Virol* 2020; 92: 2050–2054.

[20] Heriyanto RS, Kurniawan A, Wijovi F, et al. The role of COVID-19 survivor status and gender towards neutralizing antibody titers 1, 2, 3 months after Sinovac vaccine administration on clinical-year medical students in Indonesia: Role of COVID-19

survivor status and gender towards neutralizing antibody titers after Sinovac vaccine administration. *International Journal of Infectious Diseases* 2021; 113: 336–338.

[21] Ciarambino T, Para O, Giordano M. Immune system and COVID-19 by sex differences and age. *Women's Health*; 17. Epub ahead of print 2021. DOI: 10.1177/17455065211022262.

[22] Kim Y, Bae JY, Kwon K, et al. Kinetics of neutralizing antibodies against SARS-CoV-2 infection according to sex, age, and disease severity. *Sci Rep*; 12. Epub ahead of print 1 December 2022. DOI: 10.1038/s41598-022-17605-1.

[23] Statsenko Y, Al Zahmi F, Habuza T, et al. Impact of Age and Sex on COVID-19 Severity Assessed From Radiologic and Clinical Findings. *Front Cell Infect Microbiol*; 11. Epub ahead of print 25 February 2022. DOI: 10.3389/fcimb.2021.777070.

Figure 1

Click here to access/download;Figure;Figure 1.tiff

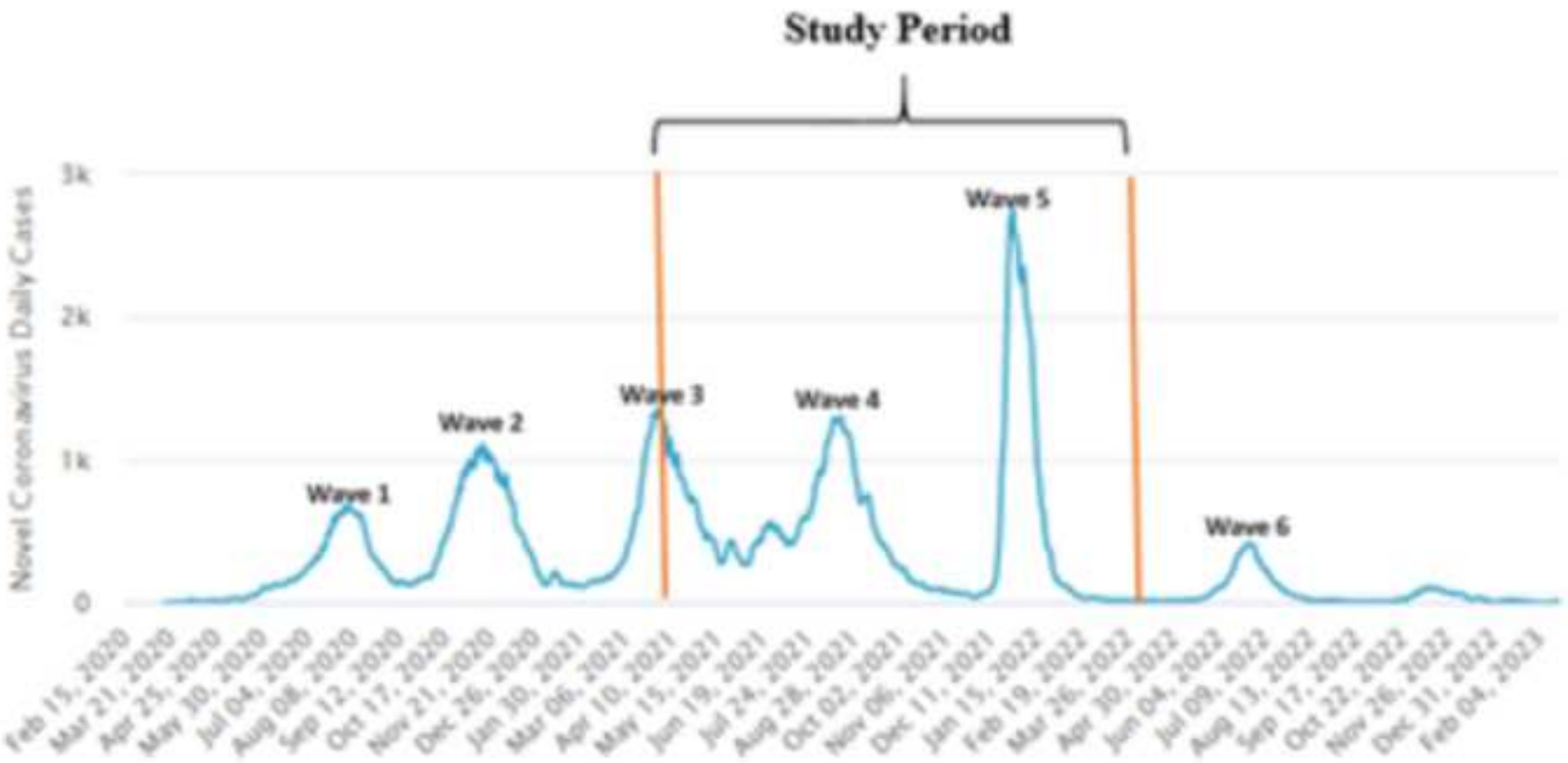

Figure 2

Click here to access/download;Figure;Figure 2.tiff

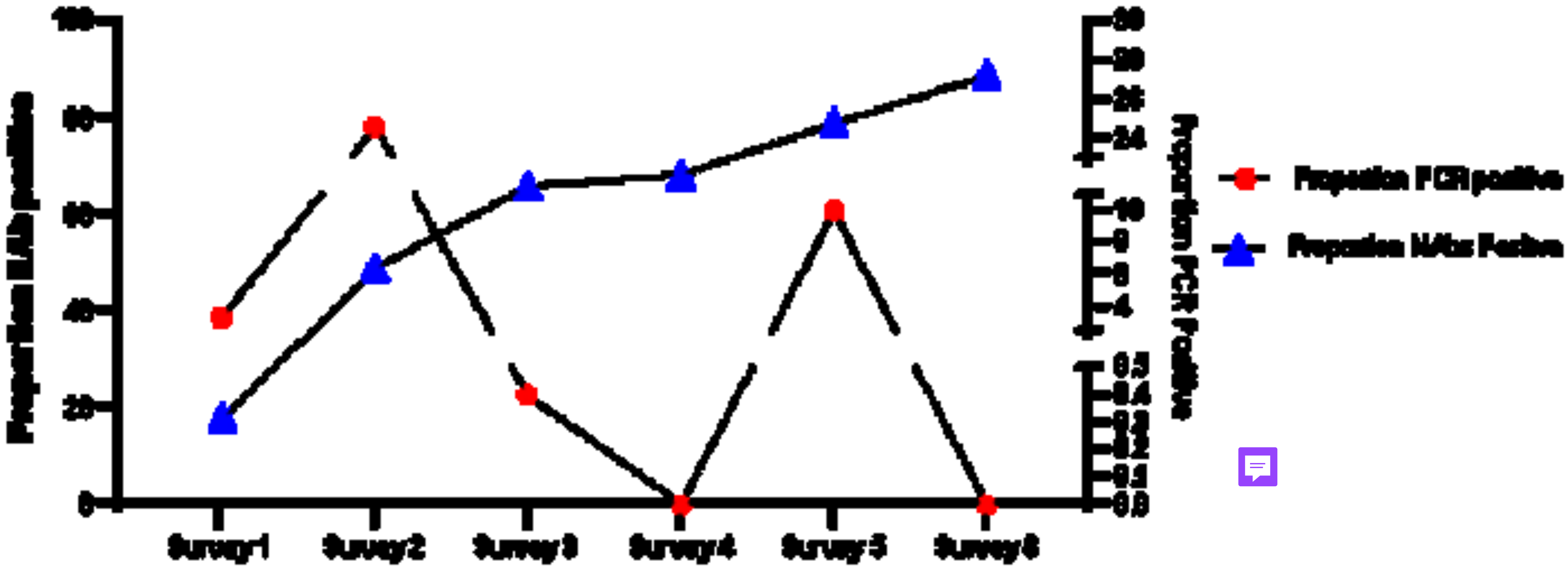

Figure 3

Click here to access/download;Figure;Figure 3.tiff

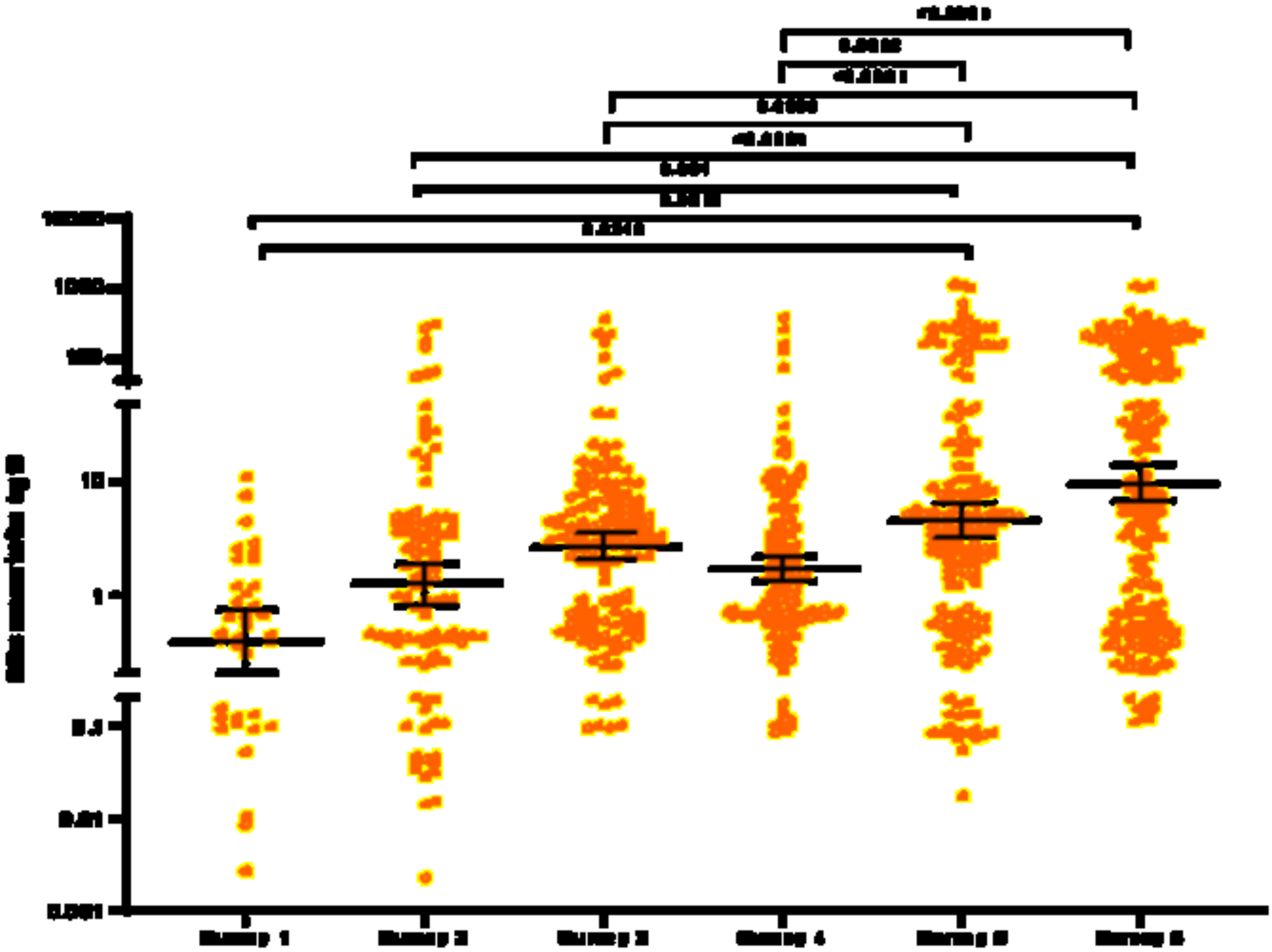

Click here to access/download;Figure;Figure 4.tiff

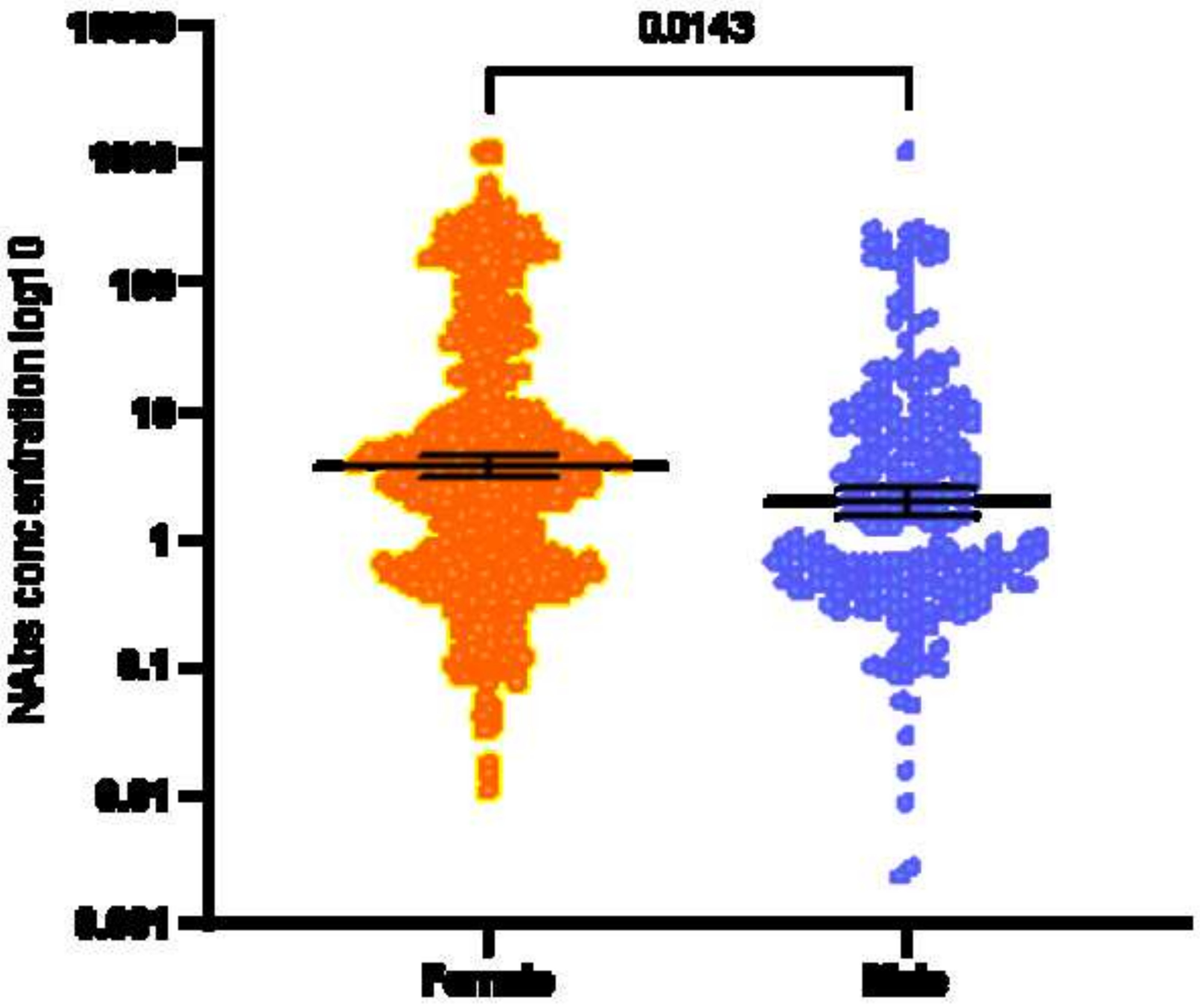

Figure 5A

Click here to access/download;Figure;Figure 5A.tiff

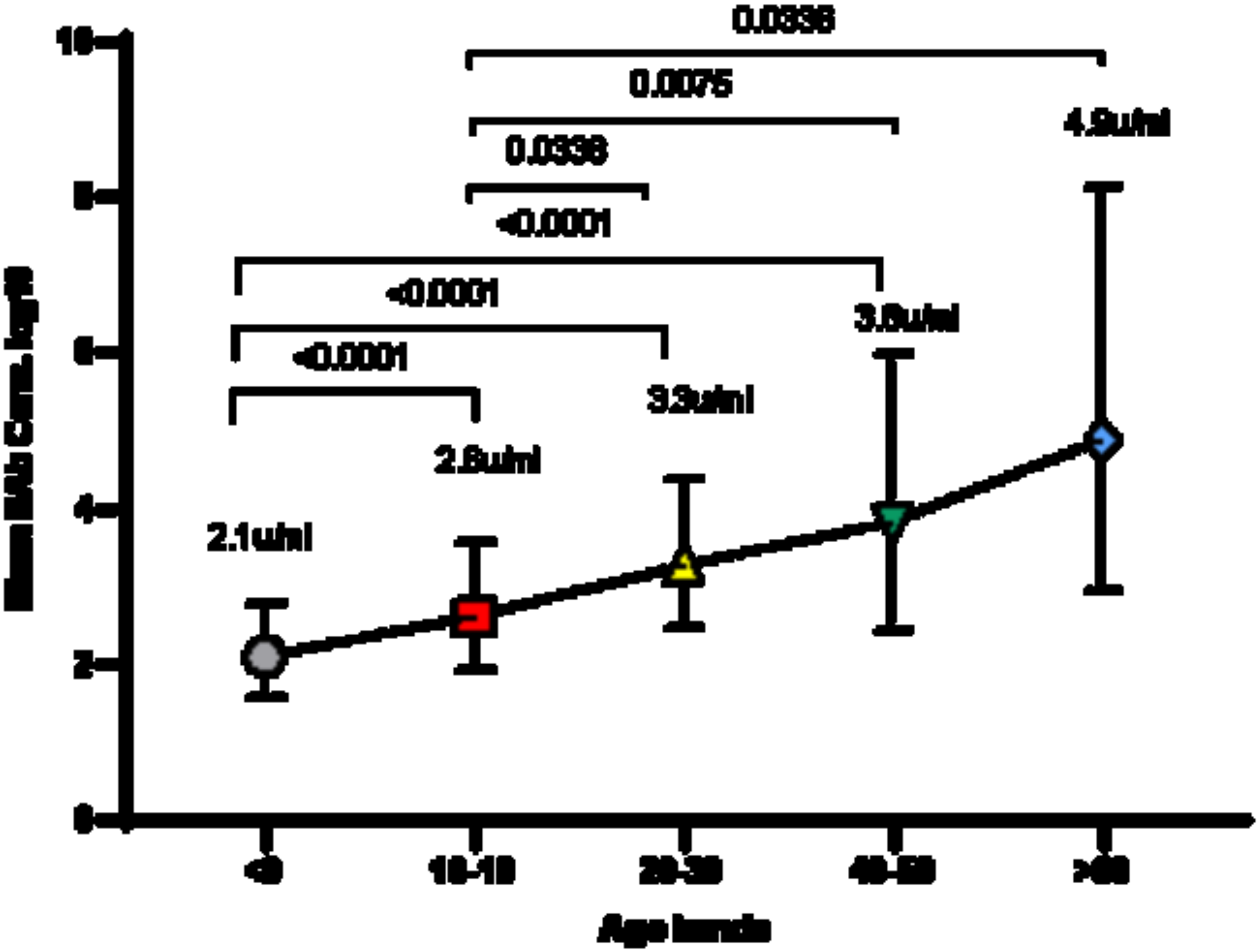

Figure 5B

Click here to access/download;Figure;Figure 5B.tiff

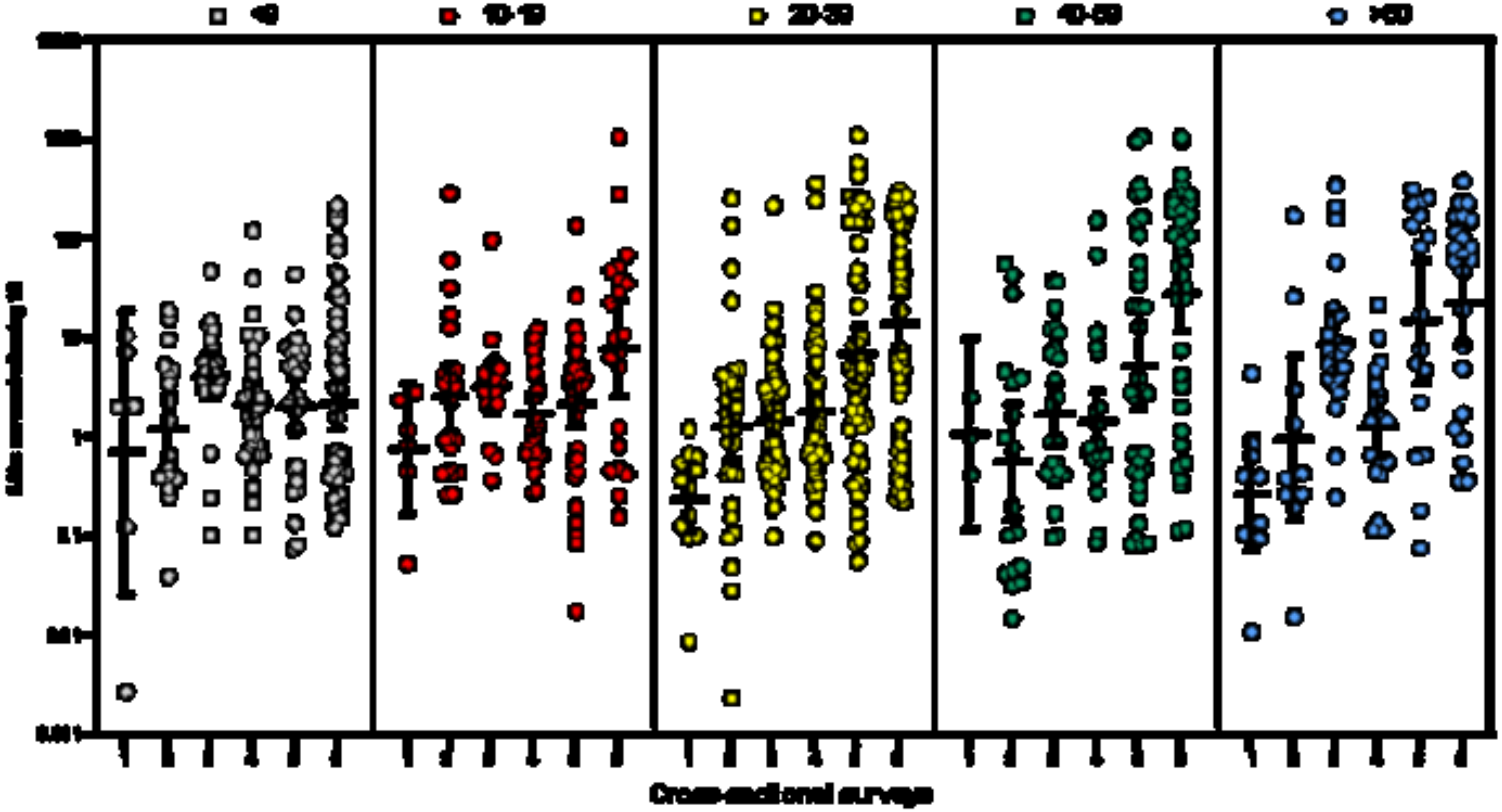

Supplement: Attachment — Submitted filename: PGPH-D-26-00167 review March 2026.pdf [file pgph.0006706.s006.pdf]
