# Supplementary material for: SARS-CoV-2 and neutralizing antibody dynamics at a remote village in Kenya before widespread COVID-19 vaccine rollout
Source: PLOS Glob Public Health. 2026 Jul 24;6(7):e0006706. doi: 10.1371/journal.pgph.0006706 (PMC13399503; doi:10.1371/journal.pgph.0006706)
Supplement: Attachment — Submitted filename: Response to Reviewers.docx [file pgph.0006706.s008.docx]

**Responses to reviewers’ comments**

1. **Reviewer comments in the body of the Editor’s email**

**Reviewer 1 Comment**

To address the research problem scientific i recommend a relook at your study design. The best approach would have been an experimental method (a longitudinal cohort study) instead of a descriptive survey study. No baseline data is available to support your claims. For instance when was COVID vaccine rolled out? This is also confirmed during your data collection, where two individuals had already received the vaccine. There are a lot of variable that can influence development or non development of herd immunity.

**Response: We thank the reviewer for these constructive comments. We agree that longitudinal cohort data can provide stronger individual‑level inference about seroconversion timing. However, we respectfully note that repeated cross‑sectional surveys were selected because they allow robust, time dependent, population‑level monitoring of seroprevalence trends when establishing and maintaining a large longitudinal prospective cohort is not feasible due to logistical, ethical, and resource constraints in this setting. We contend that repeated cross‑sectional surveillance is an accepted method for tracking changing immunity over time. We have included the justification for conducting repeated cross-sectional surveillance. See lines 121-126 in the MS with tracked changes.**

**We have also removed language implying causality or that herd immunity was observed; instead, we now describe an observed increase in population‑level neutralizing antibodies consistent with widespread exposure during the study period. We have expanded the Limitations to acknowledge unmeasured variables that could influence population immunity and note that our design cannot fully disentangle all contributors at the individual level. See Abstract lines 13-15 and lines 39-42. Also in the Discussion, lines 354-359 in the MS with tracked changes.**

**Concerning baseline data, our first survey (April 2021) provides baseline seroprevalence and PCR positivity for the study period. As for the use of the vaccine, the HDSS population is monitored routinely at individual household level and data on vaccinations, illness, migration, births and deaths collected. So, we are confident that there was minimal vaccination for COVID-19, apart from the individuals referred to in the manuscript. This information is now provided in the Introduction, Lines 108-110 in the MS with tracked changes.**

**Reviewer 2 comments**

**Comment 1**

Find comments and suggestions attached below.

Strengths

The study addresses an important public health question regarding natural immunity in under-vaccinated populations, and conducting six surveys allows for meaningful observation of temporal trends in both infection and antibody levels. Measuring neutralizing antibodies (rather than total antibodies alone) strengthens the biological relevance of the findings and data from a rural, likely underrepresented population adds valuable epidemiological insight.

Major Study Concerns: Although multiple time points were assessed, the study is cross-sectional, not longitudinal. It is unclear whether the same individuals were followed throughout. This limits the ability to conclude Individual-level immune dynamics and true “buildup” of immunity over time. The author needs to clarify participant overlap across surveys or temper conclusions regarding temporal immune acquisition.

**Response: We thank the reviewer for these constructive comments. Reviewer 1 had similar concerns. We agree that longitudinal cohort data can provide stronger individual‑level inference about seroconversion timing. However, we respectfully note that repeated cross‑sectional surveys were selected because they allow robust, timely, population‑level monitoring of seroprevalence trends when establishing and maintaining a large longitudinal prospective cohort is not feasible due to logistical, ethical, and resource constraints in this setting. We contend that repeated cross‑sectional surveillance is an accepted method for tracking changing immunity over time.**

**Nevertheless, we have removed language implying causality or that herd immunity was achieved; instead, we now describe an observed increase in population‑level neutralizing antibodies consistent with widespread exposure during the study period. We have expanded the Limitations to acknowledge unmeasured variables that could influence population immunity (e.g., differential exposure, waning immunity) and note that our design cannot fully disentangle all contributors at the individual level. See Abstract lines 13-15 and lines 39-42. Also in the Discussion, lines 354-359 in the MS with tracked changes.**

**Comment 2:**

The conclusion that herd immunity resulted solely from natural infection is not fully supported by data. No direct verification of vaccination status and a lack of anti-nucleocapsid (N) antibody testing, which could distinguish infection from vaccination. The claim of “disease-mediated herd immunity” is therefore overstated, and I would recommend revising conclusions to reflect association rather than causation.

**Response: We agree that the lack of anti-nucleocapsid (N) antibody testing, which could distinguish infection from vaccination is a limitation. As noted above, we have removed language implying causality. Instead, we now describe an observed increase in population‑level neutralizing antibodies consistent with widespread exposure during the study period. Nevertheless, the HDSS population that was studied in respect to population‑level neutralizing antibodies buildup is monitored routinely at individual household level and collect data on vaccinations, illness, migration, births and deaths. So, we are confident that there was minimal vaccination for COVID-19, apart from the individuals referred to in the manuscript. In the Discussion, lines 359-363, we have admitted that the lack of anti-nucleocapsid (N) antibody testing, which could distinguish infection from vaccination is a limitation.**

**Comment 3:**

The study uses a surrogate virus neutralization test (sVNT). While practical, sVNT measures inhibition of RBD–ACE2 binding, not true viral neutralization. This is not equivalent to gold-standard PRNT. The interpretation of “neutralizing capacity” should be made cautiously. Recommend explicitly acknowledging the limitations of sVNT in measuring functional immunity.

**Response: We acknowledge that sVNT infers neutralization by blocking RBD–ACE2 interaction and does not measure virus replication/entry in cells as PRNT does. We have added text acknowledging this limitation. We chose sVNT because it allows safe, high-throughput assessment of RBD–ACE2 blocking antibodies in a BSL-2 setting. Importantly, sVNT has been shown to correlate strongly with live-virus neutralization assays in multiple published reports (refs 30 - 32). Nevertheless, we revised the manuscript to replace ambiguous phrases such as “neutralizing capacity” with “RBD–ACE2 binding inhibition (surrogate neutralization)” and added the following limitation to the Discussion: “sVNT measures inhibition of RBD–ACE2 binding and does not directly measure viral replication or entry in permissive cells. The results should therefore be interpreted as surrogate indicators of neutralizing activity. See Discussion section, line 300 in the MS with tracked changes.**

**Comment 4:**

Changes in infection rates are attributed to rising immunity, but other confounding factors are not considered, such as Behavioral changes, Public health interventions, Testing variability, and variant-specific transmissibility. This weakens causal interpretation.

**Response: We agree that multiple time-varying factors besides population immunity can influence observed infection rates and therefore the observed rise in sVNT-measured RBD–ACE2 inhibition does not prove an immunity-associated effect. We have revised the manuscript to:**

**(1) Explicitly acknowledge sVNT’s surrogate nature. See Discussion section, lines 298-299 in the MS with tracked changes.**

**(2) Describe other potential confounders (behavioral change, non-pharmaceutical interventions, and potential cross‑reactive immunity from other coronaviruses: See in the Discussion, lines 356-359 in the MS with tracked changes.**

**(3) Tempered causal language and say that the results should be interpreted as surrogate indicators of neutralizing activity. See Discussion section, line 300 in the MS with tracked changes.**

**Comment 5:**

Description of the ELISA procedure is detailed but could be streamlined for clarity. Some technical repetition is present.

**Response: Thank you for noticing this. We have rewritten the ELISA assay. See lines 163-179 in the MS with tracked changes.**

**Comment 6**

Minor grammatical errors and inconsistencies. Some sentences are overly long or unclear. Would recommend careful proofreading and language editing.

**Response: We have proofread and re-check grammar**

**Comment 7:**

Conclusion

The study presents a valuable dataset with important public health implications, particularly in understanding SARS-CoV-2 transmission dynamics in under-vaccinated populations. However, the manuscript would benefit from a more cautious interpretation of the findings, a clearer and more comprehensive acknowledgment of methodological limitations, and a refinement of conclusions regarding the development and attribution of herd immunity.

**Response: We agree. As noted in response to comment 1, we have removed language implying causality or that herd immunity was achieved. We have acknowledged methodological limitations and note that our study design cannot fully disentangle all contributors at the individual level.**

2. **Reviewer comments in the marked copy of the Manuscript**

**General Comment**

This was a good study area that can inform how different population respond to infectious diseases in order to establish herd immunity. Historically, herd immunity is an immunological principle that has protected the vulnerable in our communities against infectious diseases. However, the method used to explore this concept is not scientifically sound. Use of experimental method would have yielded better findings that can be generalized. I recommend the authors to re-look at their study approach and make changes before consideration.

**Response: Thank you for the thoughtful review. We agree that terminology and interpretation require clarification. We respectfully note that experimental exposure studies for SARS‑CoV‑2 are unethical and impractical; therefore, repeated cross‑sectional surveillance was appropriate and is a widely used observational method to monitor population‑level transmission and immunity over time. We have included the justification for conducting repeated cross-sectional surveillance. See lines 121-126 in the MS with tracked changes.**

**Nevertheless, we have removed language implying causality or that herd immunity was achieved; instead, we now describe an observed increase in population‑level neutralizing antibodies consistent with widespread exposure during the study period. We have expanded the Limitations to acknowledge unmeasured variables that could influence population immunity and note that our design cannot fully disentangle all contributors at the individual level.**

**Specifically, we have made the following changes to address the reviewer’s concerns and to temper our conclusions:**

**-	Replaced any wording implying that the study “established herd immunity” with qualified language throughout the abstract, results, and discussion. New phrasing emphasizes an observed “buildup of population‑level neutralizing antibodies consistent with exposure”. See Abstract lines 13-15 and lines 38-42. Also in the Discussion, lines 354 - 359 in the MS with tracked changes.**

**-	Added a brief rationale for the method used. In the Method section, we say: repeated cross‑sectional surveys were chosen because they allow	serial population-level assessment when longitudinal follow-up of all individuals is not feasible, and because experimental approaches are neither ethical nor feasible for infectious exposure. See Methods section, lines 121-126 in the MS with tracked changes.**

**-	Rewrote parts of the Discussion to frame findings as informative for understanding how population‑level NAb acquisition may have evolved in a low‑vaccine‑coverage rural African setting. See Discussion, lines 262-272 in the MS with tracked changes.**

**In addition, we have revised the title to: *“SARS-CoV-2 and neutralizing antibody dynamics at a remote village in Kenya before widespread COVID-19 vaccine rollout”* to better contextualize the study period.**

**ABSTRACT**

**Reviewer:** Insert a brief background.

**Comment:** **Done. See lines 13-15.**

**Reviewer:** Replace serum with a blood sample.

**Comment:** **Done. see lines 25-26**

**Reviewer:** Put n in the brackets for easier interpretation e.g. 69% (n/1329). Do the same for the rest of the results.

**Comment: Thank you for this helpful suggestion. We have revised to include “n” values alongside percentages throughout the Abstract and manuscript.**

**Reviewer:** Provide the period for the surveys.

**Comment: Done. See lines 29 to 31.**

**Reviewer:** To be precise use the actual dates

**Comment:** **Done. See lines 29 to 31.**

**Reviewer:** What were the findings from the naso-pharyngeal swabs? It be nice if the RT-PCR findings and NABs are addressed side by side.

**Comment:** **Thank you for raising this concern. We have revised the abstract in lines 28 – 32 to read: “By Real Time PCR, SARS-CoV-2 increased from 3.1% (7/224) at survey one, April 2021 to 24.0% (55/229) by month two, and thereafter decreased to zero, only to rise to 10% (23/234) during the Omicron wave, January 2022. Participants with NAbs increased from 18% (40/224) at survey one to 86% (202/234) by end of the study”.**

**Point 8**

**Reviewer:** What was the age distribution?

**Comment: We thank the reviewer for raising this concern. This has been clearly highlighted in the abstract. See lines 35 – 36.**

**Point 9**

**Reviewer:** What data support this claim? Survey as a method cannot establish causation.

**Comment:**

**We acknowledge that causality cannot be inferred from a cross-sectional study design. The statement has been revised accordingly to reflect population-level observations. Given that the study was conducted prior to widespread vaccine rollout in the study area, the observed increase in neutralizing antibodies is most consistent with natural infection-driven exposure rather than vaccination. We have removed causal wording. See lines 38 - 39.**

**INTRODUCTION**

**Point 10**

**Reviewer:** Include the 'Why' of this study. What was the research problem? being addressed. Build your background using the funnel approach to help readers understand the context of this study. Let us see how you conceptualize ideas and your thought process.

**Comment:**

**Thank you for this important comment. We have revised the Introduction using a funnel approach to better articulate the background, research problem, and rationale for the study. The revised version now starts with the global and regional burden of COVID-19, narrows down to immunological considerations and measurement challenges, and clearly identifies the gap in knowledge regarding community-level infection and antibody dynamics in rural African settings in lines 85 - 93. We have also stated the “why” of the study and strengthened the conceptual flow to better guide the reader to the study objective. See lines 53 - 55 and lines 94 - 98.**

**Point 11**

**Reviewer:** What was the objective of this study?

**Comment:**

**We have clarified the objective of the study in the revised manuscript for improved readability and alignment with the study design. The objective was to characterize temporal patterns of SARS-CoV-2 infection using Real Time PCR and neutralizing antibody responses using a surrogate virus neutralization test in a rural, largely unvaccinated population in western Kenya prior to widespread COVID-19 vaccine rollout, using cross-sectional surveys within the Kombewa Health and Demographic Surveillance System (HDSS). See lines 94 - 98**

**MATERIALS AND METHODS**

**Point 12**

**Reviewer:** Why Kombewa?

**Comment:**

**We have clarified the rationale for selecting the Kombewa study site in the revised manuscript under the “Study Site and Population” section page, lines 108 - 110. Kombewa was chosen because it hosts the WRAIR-Africa Health and Demographic Surveillance System (HDSS), which provides a well-established rural surveillance platform with a defined population of approximately 160,000 individuals across about 30,000 households. The HDSS provides routinely updated and high-quality demographic data, making it suitable for population-based infectious disease surveillance. In addition, the predominantly rural setting and existing data infrastructure made it an ideal site for assessing SARS-CoV-2 transmission and antibody dynamics across all age groups and both genders.**

**Point 13**

**Reviewer:** Cite source of this information. “about 160,000 inhabitants who live

68 in about 30,000 households”

**Comment: Added the reference [20], line 106.**

**Point 14**

**Reviewer:** What was your inclusion and exclusion criteria?

**Comment:**

**We have now added a clear section on eligibility criteria in the revised manuscript. See lines 140-145. The inclusion criteria comprised all individuals of any age who resided within the HDSS and were identified for recruitment into the study. The exclusion criteria included refusal to provide informed consent and contraindication to venipuncture.**

**Point 15**

**Reviewer:** What about history of COVID ?

**Comment:**

**Thank you for this comment. We acknowledge that self-reported history of prior COVID-19 infection was not collected in this study. The study instead relied on RT-PCR testing at each survey round to detect active infection and serological assessment of neutralizing antibodies as a proxy for prior exposure.**

**Point 16**

**Reviewer:** This is contradictory. When and where did they get the vaccine? Does this resonate with the study title?

**Comment:**

**Thank you for this important comment. We have removed the statement regarding the number of vaccinated participants to avoid ambiguity, (The last sentence under the section on Study site and population (lines 111-112). Vaccination status was collected as background information but was not a primary variable in the analysis. The manuscript has been revised to ensure consistency with the study objective and title, which focuses on SARS-CoV-2 infection and NAb dynamics prior to widespread vaccine rollout.**

**Point 17**

**Reviewer:** What was your study design ? cross sectional or a longitudinal ?

Describe your sampling design and give a rationale. How was your sample size calculated?

**Comment:**

**Thank you for this important comment. We have revised the manuscript to clearly clarify the study design, sampling strategy, and sample size calculation.**

**The study employed a cross-sectional study design conducted through six survey rounds spaced two months apart over a 12-month period within the Kombewa HDSS population. At each survey round, a new cross-sectional sample of households and participants was randomly selected by the HDSS data manager, ensuring representation across age bands (See lines 120-124).**

**Regarding sampling, the HDSS manager identified clusters of households that had appropriate age band categories and individuals invited to participate in the study (see lines 124 to 130).**

**We have also clarified the sample size calculation (see lines 132-139). The sample size for each survey round was estimated using a single population proportion formula based on expected seroprevalence, with an assumed prevalence of 2.5% and a tolerated margin of error of 2% at a 95% confidence level. This yielded a target sample size of 234 participants per survey round, giving a total of 1,404 observations across six surveys. The assumed prevalence was based on the early phase of the pandemic when seropositivity was expected to be minimal in the study setting.**

**RESULTS**

**Point 18**

**Reviewer:** Apart from Age and gender were there any other variable of interest for this study? You mentioned in your method that the manager identified the households, what were the characteristics of this households. Could this have led to sampling bias between male and female?

**Comment:**

**Thank you for this observation. The primary variables of interest for this study were SARS-CoV-2 infection status, neutralizing antibody responses, age, and sex. The HDSS manager identified clusters of households that had representative demographics (age and gender) and individuals invited to participate in the study. We acknowledge that the higher proportion of female participants may reflect availability at home during the surveys. While this may have introduced participation bias, it cannot explain the high proportion of seropositivity, nor the NAb titers observed in females. We have added this information to the Discussion. See lines 319 to 331.**

**Point 19**

**Reviewer:** Use detected instead of positivity for RT-PCR results. Use actual months instead of survey one....etc. Were this participants cohort?

**Comment:**

**Thank you for this important comment. The manuscript has been revised to replace “positivity” with “detected” for RT-PCR results and to report sampling time points using actual calendar months rather than survey numbering for clarity (lines 207 – 218). We also clarify that the study was repeated cross-sectional surveys within the HDSS population (lines 120-124).**

**Point 20**

**Reviewer:** Was sequencing done on the samples?

**Comment: This statement has been removed from Figure 2, legend. VOC were inferred from the corresponding National waves, shown in Figure 1.**

**Point 21**

**Reviewer:** Was this concentration from the same participants?

**Comment: Each survey round included independent participants drawn from the study population. This information is now included. See lines 224-225**

**Point 22**

**Reviewer:** Were this values from pooled concentration?

**Comment:**

**The gender values were performed individually but analyzed as a pool for females and males. We also performed stratified analyses at each survey to assess temporal variation (see Figure 4B). Both pooled and survey-specific estimates are now provided.**

**CONCLUSION**

**Point 23**

**Reviewer:** Longitudinal cohort design would have been the best method. A lot of confounders were not put into consideration in this study. Therefore, this conclusion cannot be conclusive. What will be your recommendation.

**Comment:**

**Response: We thank the reviewer for these constructive comments. We agree that longitudinal cohort data can provide stronger individual‑level inference about seroconversion timing. However, we respectfully note that repeated cross‑sectional surveys were selected because they allow robust, timely, population‑level monitoring of seroprevalence trends when establishing and maintaining a large longitudinal prospective cohort is not feasible due to logistical, ethical, and resource constraints in this setting. We contend that repeated cross‑sectional surveillance is an accepted method for tracking changing immunity over time.**

**Nevertheless, we have removed language implying causality or that herd immunity was achieved; instead, we now describe an observed increase in population‑level neutralizing antibodies consistent with widespread exposure during the study period. We have expanded the Limitations to acknowledge unmeasured variables that could influence population immunity (e.g., differential exposure, waning immunity) and note that our design cannot fully disentangle all contributors at the individual level. See Abstract lines 13-15 and lines 39-42. Also in the Discussion, lines 354-359 in the MS with tracked changes.**
