# Supplementary material for: SARS-CoV-2 and neutralizing antibody dynamics at a remote village in Kenya before widespread COVID-19 vaccine rollout
Source: PLOS Glob Public Health. 2026 Jul 24;6(7):e0006706. doi: 10.1371/journal.pgph.0006706 (PMC13399503; doi:10.1371/journal.pgph.0006706)
Supplement: Peer review history

# Peer review: Decision Letter 0

**DOI:** 10.1371/journal.pgph.0006706.r001

**Authors:** Abram Wagner

13 Apr 2026

PGPH-D-26-00167

SARS-CoV-2 and neutralizing antibody dynamics at a remote village in Kenya before COVID-19 vaccine rollout

PLOS Global Public Health

Dear Dr. Waitumbi,

Thank you for submitting your manuscript to PLOS Global Public Health. After careful consideration, we feel that it has merit but does not fully meet PLOS Global Public Health’s publication criteria as it currently stands. Therefore, we invite you to submit a revised version of the manuscript that addresses the points raised during the review process.

Both reviewers had major concerns about your interpretations - please make sure that this is interpreted carefully as a survey instead of as a cohort or as an RCT (which could have causal implications). Note that Reviewer 1 has comments in an attached document. It is ok if not all comments are addressed if you feel like they are not relevant or if they conflict across reviewers - just let me know your reasoning.

Please submit your revised manuscript by May 28 2026 11:59PM. If you will need more time than this to complete your revisions, please reply to this message or contact the journal office at globalpubhealth@plos.org. When you're ready to submit your revision, log on to https://www.editorialmanager.com/pgph/ and select the 'Submissions Needing Revision' folder to locate your manuscript file.

Please include the following items when submitting your revised manuscript:

- A letter that responds to each point raised by the editor and reviewer(s). You should upload this letter as a separate file labeled 'Response to Reviewers'.
- A marked-up copy of your manuscript that highlights changes made to the original version. You should upload this as a separate file labeled 'Revised Manuscript with Track Changes'.
- An unmarked version of your revised paper without tracked changes. You should upload this as a separate file labeled 'Manuscript'.

Guidelines for resubmitting your figure files are available below the reviewer comments at the end of this letter.

As the corresponding author, your ORCID iD is verified in the submission system and will appear in the published article. PLOS supports the use of ORCID, and we encourage all coauthors to register for an ORCID iD and use it as well. Please encourage your coauthors to verify their ORCID iD within the submission system before final acceptance, as unverified ORCID iDs will not appear in the published article. *Only*  the individual author can complete the verification step; PLOS staff *cannot*  verify ORCID iDs on behalf of authors.

We look forward to receiving your revised manuscript.

Kind regards,

Abram L. Wagner, PhD, MPH

Academic Editor

PLOS Global Public Health

Journal Requirements:

1. Please send a completed 'Competing Interests' statement, including any COIs declared by your co-authors. If you have no competing interests to declare, please state "The authors have declared that no competing interests exist".

1. Please amend your detailed Financial Disclosure statement. This is published with the article. It must therefore be completed in full sentences and contain the exact wording you wish to be published.
  If you did not receive any funding for this study, please simply state: “The authors received no specific funding for this work.”

1. We note that your Data Availability Statement is currently as follows: “All the data are available in the manuscript”.
  Please confirm at this time whether or not your submission contains all raw data required to replicate the results of your study. Authors must share the “minimal data set” for their submission. PLOS defines the minimal data set to consist of the data required to replicate all study findings reported in the article, as well as related metadata and methods (https://journals.plos.org/plosone/s/data-availability#loc-minimal-data-set-definition).
  For example, authors should submit the following data:
  - The values behind the means, standard deviations and other measures reported;
  - The values used to build graphs;
  - The points extracted from images for analysis.
  Authors do not need to submit their entire data set if only a portion of the data was used in the reported study.
  If your submission does not contain these data, please either upload them as Supporting Information files or deposit them to a stable, public repository and provide us with the relevant URLs, DOIs, or accession numbers. For a list of recommended repositories, please see https://journals.plos.org/plosone/s/recommended-repositories.
  If there are ethical or legal restrictions on sharing a de-identified data set, please explain them in detail (e.g., data contain potentially sensitive information, data are owned by a third-party organization, etc.) and who has imposed them (e.g., an ethics committee). Please also provide contact information for a data access committee, ethics committee, or other institutional body to which data requests may be sent. If data are owned by a third party, please indicate how others may request data access.

If the reviewer comments include a recommendation to cite specific previously published works, please review and evaluate these publications to determine whether they are relevant and should be cited. There is no requirement to cite these works unless the editor has indicated otherwise.

Additional Editor Comments (if provided):

Can you provide more high res versions of your figures?

[Note: HTML markup is below. Please do not edit.]

Reviewers' comments:

Reviewer's Responses to Questions

**Comments to the Author**

1. Does this manuscript meet PLOS Global Public Health’s publication criteria? Is the manuscript technically sound, and do the data support the conclusions? The manuscript must describe methodologically and ethically rigorous research with conclusions that are appropriately drawn based on the data presented.

Reviewer #1: No

Reviewer #2: Yes

**********

2. Has the statistical analysis been performed appropriately and rigorously?

Reviewer #1: No

Reviewer #2: Yes

**********

3. Have the authors made all data underlying the findings in their manuscript fully available (please refer to the Data Availability Statement at the start of the manuscript PDF file)?

The PLOS Data policy requires authors to make all data underlying the findings described in their manuscript fully available without restriction, with rare exception. The data should be provided as part of the manuscript or its supporting information, or deposited to a public repository. For example, in addition to summary statistics, the data points behind means, medians and variance measures should be available. If there are restrictions on publicly sharing data—e.g. participant privacy or use of data from a third party—those must be specified.

Reviewer #1: No

Reviewer #2: Yes

**********

4. Is the manuscript presented in an intelligible fashion and written in standard English?

PLOS Global Public Health does not copyedit accepted manuscripts, so the language in submitted articles must be clear, correct, and unambiguous. Any typographical or grammatical errors should be corrected at revision, so please note any specific errors here.

Reviewer #1: Yes

Reviewer #2: Yes

**********

5. Review Comments to the Author

Please use the space provided to explain your answers to the questions above. You may also include additional comments for the author, including concerns about dual publication, research ethics, or publication ethics. (Please upload your review as an attachment if it exceeds 20,000 characters)

Reviewer #1: To address the research problem scientific i recommend a relook at your study design. The best approach would have been an experimental method (a longitudinal cohort study) instead of a descriptive survey study. No baseline data is available to support your claims. For instance when was COVID vaccine rolled out? This is also confirmed during your data collection, where two individualshad already received the vaccine. There are a lot of variable that can influence development or non development of herd immunity.

Reviewer #2: Dear Author,

Find comments and suggestions attached below.

Strengths

The study addresses an important public health question regarding natural immunity in under-vaccinated populations, and conducting six surveys allows for meaningful observation of temporal trends in both infection and antibody levels. Measuring neutralizing antibodies (rather than total antibodies alone) strengthens the biological relevance of the findings and data from a rural, likely underrepresented population adds valuable epidemiological insight.

Major Study Concerns: Although multiple time points were assessed, the study is cross-sectional, not longitudinal. It is unclear whether the same individuals were followed throughout. This limits the ability to conclude Individual-level immune dynamics and true “buildup” of immunity over time. The author needs to clarify participant overlap across surveys or temper conclusions regarding temporal immune acquisition.

The conclusion that herd immunity resulted solely from natural infection is not fully supported by data. No direct verification of vaccination status and a lack of anti-nucleocapsid (N) antibody testing, which could distinguish infection from vaccination. The claim of “disease-mediated herd immunity” is therefore overstated, and I would recommend revising conclusions to reflect association rather than causation.

The study uses a surrogate virus neutralization test (sVNT). While practical, sVNT measures inhibition of RBD–ACE2 binding, not true viral neutralization. This is not equivalent to gold-standard PRNT. The interpretation of “neutralizing capacity” should be made cautiously. Recommend explicitly acknowledging the limitations of sVNT in measuring functional immunity.

Changes in infection rates are attributed to rising immunity, but other confounding factors are not considered, such as Behavioral changes, Public health interventions, Testing variability, and variant-specific transmissibility. This weakens causal interpretation.

Description of the ELISA procedure is detailed but could be streamlined for clarity. Some technical repetition is present.

Minor grammatical errors and inconsistencies. Some sentences are overly long or unclear. Would recommend careful proofreading and language editing.

Conclusion

The study presents a valuable dataset with important public health implications, particularly in understanding SARS-CoV-2 transmission dynamics in under-vaccinated populations. However, the manuscript would benefit from a more cautious interpretation of the findings, a clearer and more comprehensive acknowledgment of methodological limitations, and a refinement of conclusions regarding the development and attribution of herd immunity.

**********

6. PLOS authors have the option to publish the peer review history of their article (what does this mean?). If published, this will include your full peer review and any attached files.

**Do you want your identity to be public for this peer review?**  If you choose “no”, your identity will remain anonymous but your review may still be made public.

For information about this choice, including consent withdrawal, please see our Privacy Policy.

Reviewer #1: **Yes:** Festus Mulakoli

Reviewer #2: No

**********

[NOTE: If reviewer comments were submitted as an attachment file, they will be attached to this email and accessible via the submission site. Please log into your account, locate the manuscript record, and check for the action link "View Attachments". If this link does not appear, there are no attachment files.]

-->Figure Resubmissions:

While revising your submission, we strongly recommend that you use PLOS’s NAAS tool (https://ngplosjournals.pagemajik.ai/artanalysis) to test your figure files. NAAS can convert your figure files to the TIFF file type and meet basic requirements (such as print size, resolution), or provide you with a report on issues that do not meet our requirements and that NAAS cannot fix.

After uploading your figures to PLOS’s NAAS tool - https://ngplosjournals.pagemajik.ai/artanalysis, NAAS will process the files provided and display the results in the "Uploaded Files" section of the page as the processing is complete. If the uploaded figures meet our requirements (or NAAS is able to fix the files to meet our requirements), the figure will be marked as "fixed" above. If NAAS is unable to fix the files, a red "failed" label will appear above. When NAAS has confirmed that the figure files meet our requirements, please download the file via the download option, and include these NAAS processed figure files when submitting your revised manuscript.

Attachment Submitted filename: PGPH-D-26-00167 review March 2026.pdf

# Author comment: Author response to Decision Letter 1

**DOI:** 10.1371/journal.pgph.0006706.r002

4 Jun 2026

Attachment Submitted filename: Response to Reviewers.docx

# Peer review: Decision Letter 1

**DOI:** 10.1371/journal.pgph.0006706.r003

**Authors:** Abram Wagner

29 Jun 2026

SARS-CoV-2 and neutralizing antibody dynamics at a remote village in Kenya before COVID-19 vaccine rollout

PGPH-D-26-00167R1

Dear Dr. Waitumbi,

We are pleased to inform you that your manuscript 'SARS-CoV-2 and neutralizing antibody dynamics at a remote village in Kenya before COVID-19 vaccine rollout' has been provisionally accepted for publication in PLOS Global Public Health.

Before your manuscript can be formally accepted you will need to complete some formatting changes, which you will receive in a follow up email. A member of our team will be in touch with a set of requests.

Please note that your manuscript will not be scheduled for publication until you have made the required changes, so a swift response is appreciated.

IMPORTANT: The editorial review process is now complete. PLOS will only permit corrections to spelling, formatting or significant scientific errors from this point onwards. Requests for major changes, or any which affect the scientific understanding of your work, will cause delays to the publication date of your manuscript.

If your institution or institutions have a press office, please notify them about your upcoming paper to help maximize its impact. If they'll be preparing press materials, please inform our press team as soon as possible -- no later than 48 hours after receiving the formal acceptance. Your manuscript will remain under strict press embargo until 2 pm Eastern Time on the date of publication. For more information, please contact globalpubhealth@plos.org.

Thank you again for supporting Open Access publishing; we are looking forward to publishing your work in PLOS Global Public Health.

Best regards,

Abram L. Wagner, PhD, MPH

Academic Editor

PLOS Global Public Health

***********************************************************

Reviewer Comments (if any, and for reference):

Reviewer's Responses to Questions

**Comments to the Author**

1. If the authors have adequately addressed your comments raised in a previous round of review and you feel that this manuscript is now acceptable for publication, you may indicate that here to bypass the “Comments to the Author” section, enter your conflict of interest statement in the “Confidential to Editor” section, and submit your "Accept" recommendation.

Reviewer #1: All comments have been addressed

Reviewer #2: All comments have been addressed

**********

2. Does this manuscript meet PLOS Global Public Health’s publication criteria? Is the manuscript technically sound, and do the data support the conclusions? The manuscript must describe methodologically and ethically rigorous research with conclusions that are appropriately drawn based on the data presented.

Reviewer #1: Yes

Reviewer #2: Yes

**********

3. Has the statistical analysis been performed appropriately and rigorously?

Reviewer #1: Yes

Reviewer #2: Yes

**********

4. Have the authors made all data underlying the findings in their manuscript fully available (please refer to the Data Availability Statement at the start of the manuscript PDF file)?

The PLOS Data policy requires authors to make all data underlying the findings described in their manuscript fully available without restriction, with rare exception. The data should be provided as part of the manuscript or its supporting information, or deposited to a public repository. For example, in addition to summary statistics, the data points behind means, medians and variance measures should be available. If there are restrictions on publicly sharing data—e.g. participant privacy or use of data from a third party—those must be specified.

Reviewer #1: Yes

Reviewer #2: Yes

**********

5. Is the manuscript presented in an intelligible fashion and written in standard English?

PLOS Global Public Health does not copyedit accepted manuscripts, so the language in submitted articles must be clear, correct, and unambiguous. Any typographical or grammatical errors should be corrected at revision, so please note any specific errors here.

Reviewer #1: Yes

Reviewer #2: Yes

**********

6. Review Comments to the Author

Please use the space provided to explain your answers to the questions above. You may also include additional comments for the author, including concerns about dual publication, research ethics, or publication ethics. (Please upload your review as an attachment if it exceeds 20,000 characters)

Reviewer #1: Under results, authors to provide findings with discussing and describing them. This should be moved to appropriate sections (Method and Discussion section). A map of the study site will help to avoid misinterpretation by readers especially on sample size calcultion.

Reviewer #2: Thank you.

All comments were addressed, and no more recommendations were made right now.

**********

7. PLOS authors have the option to publish the peer review history of their article (what does this mean?). If published, this will include your full peer review and any attached files.

**Do you want your identity to be public for this peer review?**  If you choose “no”, your identity will remain anonymous but your review may still be made public.

For information about this choice, including consent withdrawal, please see our Privacy Policy.

Reviewer #1: **Yes:** Dr Festus Mulakoli

Reviewer #2: No

**********
